# Supplementary material for: Inhibitory Kcnip2 neurons of the spinal dorsal horn control behavioral sensitivity to environmental cold
Source: Neuron. 2023 Jan 4;111(1):92–105.e5. doi: 10.1016/j.neuron.2022.10.008 (PMC9831669; doi:10.1016/j.neuron.2022.10.008)
Supplement: Document S1. Figures S1–S9 [file mmc1.pdf]

Neuron, Volume *111*

Supplemental information

Inhibitory *Kcnip2* neurons of the spinal dorsal horn control behavioral sensitivity to environmental cold

Gioele W. Albisetti, Robert P. Ganley, Francesca Pietrafesa, Karolina Werynska, Marília Magalhaes de Sousa, Rebecca Sipione, Louis Scheurer, Michael R. Bösl, Pawel Pelczar, Hendrik Wildner, and Hanns Ulrich Zeilhofer

Inhibitory *Kcnip2* neurons of the spinal dorsal horn control behavioral sensitivity to environmental cold

Gioele W. Albisetti, Robert Ganley, Francesca Pietrafesa, Karolina Werynska, Marília Magalhaes de Sousa, Rebecca Sipione, Louis Scheurer, Michael R. Bösl, Pawel Pelczar, Hendrik Wildner, Hanns Ulrich Zeilhofer

Supplemental figures

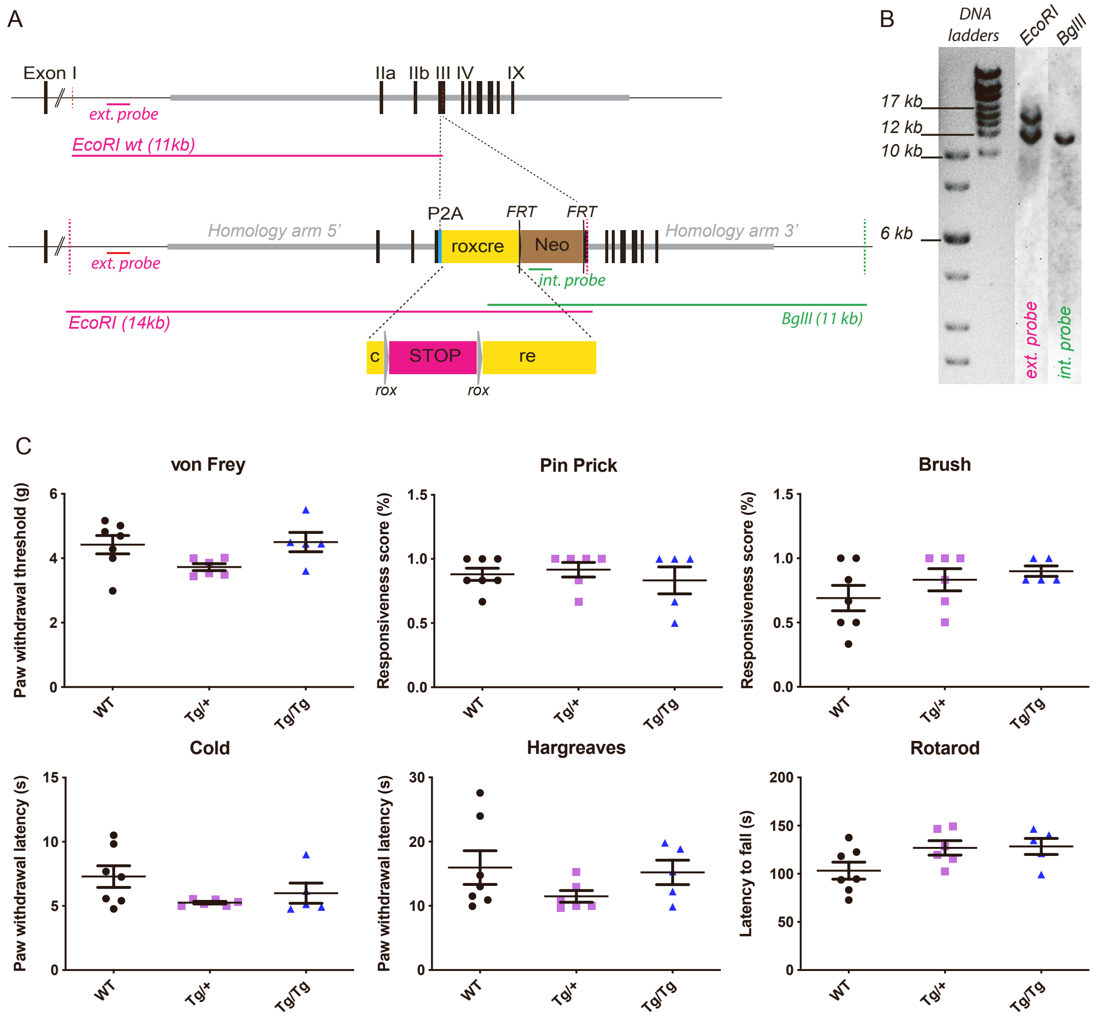

Fig. S1: Generation of *Kcnip2*^{roxCre} knock-in mice, Related to Figure 1 and STAR Methods. A, Scheme showing the targeting strategy for the generation of *Kcnip2*^{roxCre} mouse line. Homologous recombination was used to insert the cloned construct containing *P2A*, *roxCre* and *Neo* sequences into exon III of *Kcnip2* gene. The *Neo* sequence has been removed in a second step through cross breeding with *ACTB:FLPe* mice (not shown). Probes, restriction sites, length of the fragments generated, and the probes used for southern blot analysis are indicated in the figure. **B**, Southern blot result obtained from a clone with correct integration of the cloned construct. gDNA digested with EcoR I restriction enzyme and hybridized using *5'ext* probe results in a labelling of two bands of 14 kb and 11 kb in length, whereas gDNA digested with Bgl II restriction enzyme and hybridized using *Neo* probe labels a single DNA fragment of 11 kb. **C**, Sensory responses in wild-type mice (n = 7) and heterozygous (n = 6) and homozygous *Kcnip2*^{roxCre} mice (n = 5). One-way ANOVA: von Frey $F(2,15) = 2.902$, $P = 0.0860$; pin prick $F(2,15) = 0.3429$, $P = 0.7151$; brush $F(2,15) = 1.576$, $P = 0.2393$, cold $F(2,15) = 2.427$, $P = 0.1221$, Hargreaves $F(2,15) = 1.379$, $P = 0.2821$; rotarod $F(2,15) = 2.993$, $P = 0.0806$. Mean ± SEM.

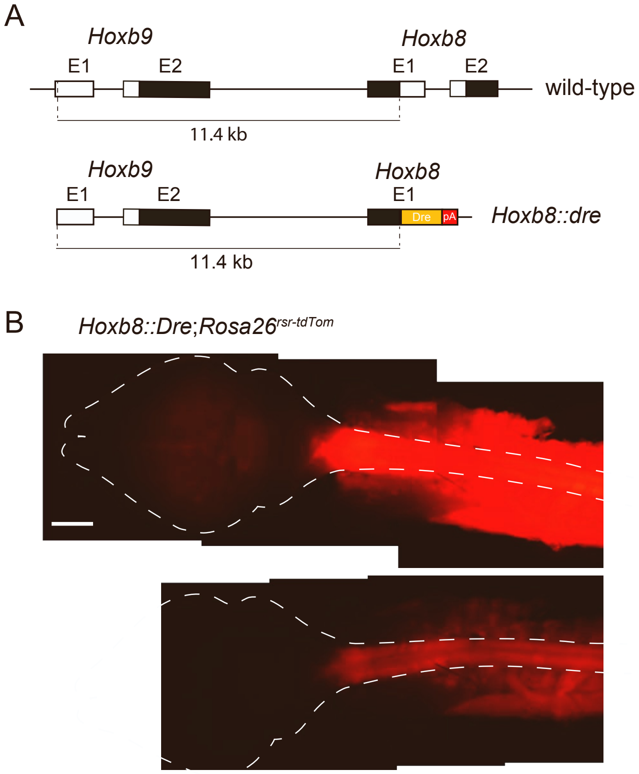

Fig. S2: Generation and gross characterization of Hoxb8::Dre BAC transgenic mice, Related to Figure 1 and STAR Methods. A, Genomic structure of the murine wild-type *HoxB8* gene locus. The 11.4 kb genomic fragment that has been used to drive expression of the Dre transgene is indicated. The *Hoxb8::Dre* transgene contains the 11.4 kb genomic fragment including the annotated *Hoxb8* promoter and a Dre expression cassette in frame with the ATG of the *Hoxb8* gene. (B,C) Expression analysis of the recombination pattern that occurred in *Hoxb8::Dre;Rosa26$^{rsr\text{-}tdTom}$* double transgenic mice. **B**, Widespread tdTom expression was observed in the CNS (outlined by dotted white line) and other tissues in the trunk of double transgenic animals. No tdTom expression was observed at supraspinal levels. Top and bottom micrographs show the same tissue but were obtained with different exposure times. Scale bars, 1 mm.

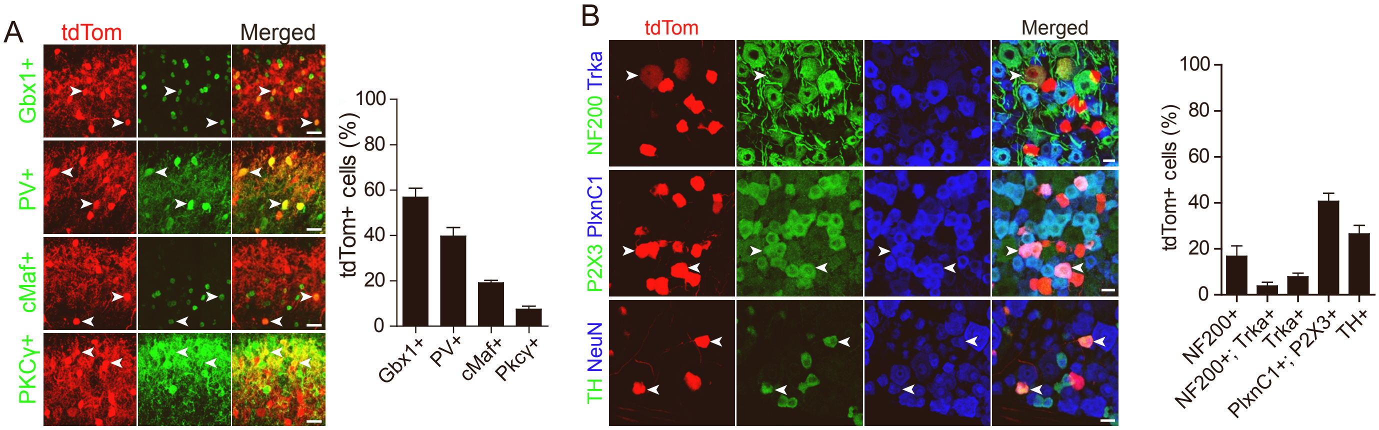

Fig. S3: Neurochemical characterization of *Kcnip2*^{Hoxb8} neurons, Related to Figure 1. Lumbar dorsal horn (A) and lumbar DRGs (B). Percent tdTom positive cells expressing the respective marker. n = 9 sections from a total of 3 mice (A) and n = 6 sections from 2 mice (B). Scale bars, 20 µm. Mean ± SEM.

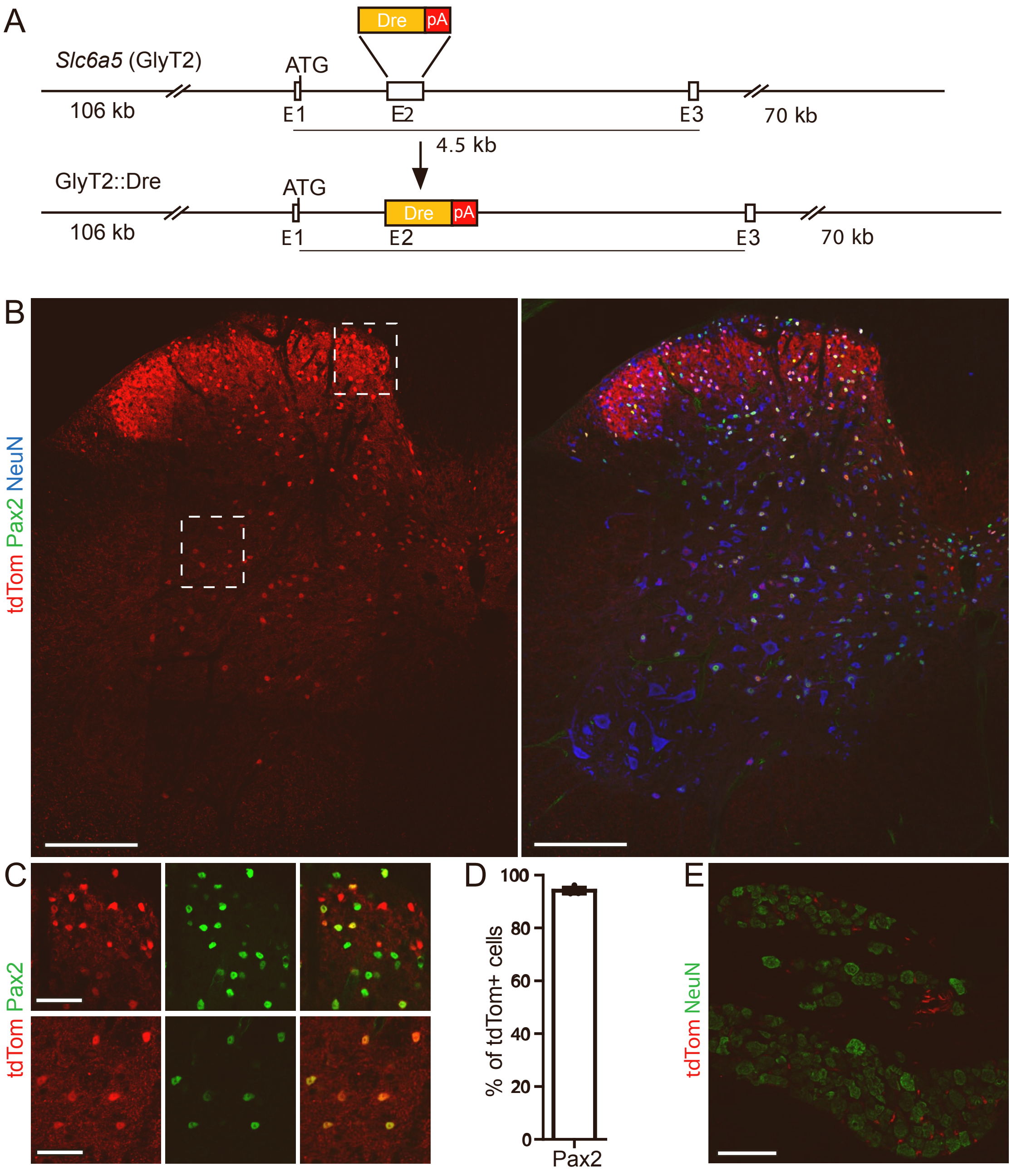

Fig. S4: Generation and characterization of BAC transgenic *GlyT2::Dre* mice, Related to Figure 2 and STAR Methods. A, Schematic representation of the generation of the *GlyT2::Dre* allele. The *GlyT2::Dre* coding sequence was placed into the second exon of the GlyT2 gene that starts with the second coding triplet. **B**, Immunohistochemistry showing the overlap of tdTom, Pax2 and NeuN in lumbar spinal cord sections of *GlyT2::Dre* mice crossed with Rosa26$^{rsr\text{-}tdTom}$ reporter mice. **C**, Insets from (B) showing the overlap between tdTom and Pax2. **D**, Quantification of (C) (913 tdTom+ neurons from n = 3 mice) **E**, No expression of tdTom in the DRG of *GlyT2::Dre* mice crossed with Rosa26$^{rsr\text{-}tdTom}$ reporter mice. Scale bars: B, 200 µm; C, 20 µm; E,100 µm. Mean ± SEM.

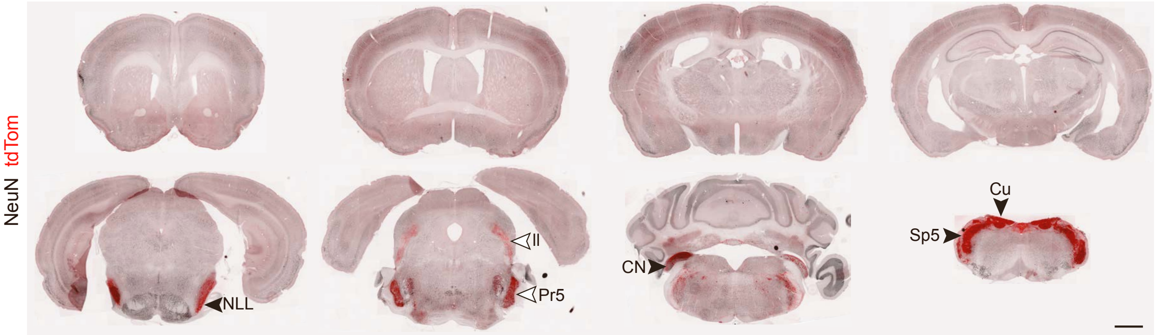

Fig. S5: Supraspinal *Kcnip2* expression, Related to Figure 1. Outside the spinal cord, *Kcnip2*^{GlyT2}-tdTom neurons were observed only in the hindbrain, including the brainstem, in particular in the spinal trigeminal nuclei (Sp5) and in the cuneate nucleus (Cu), which receive somatosensory information from the face and upper body, respectively. In addition, neurons were detected along the auditory pathways in the cochlear nucleus (CN) and in the nuclei of the lateral lemniscus (NLL), which are structures that receive and process acoustic information coming from the inner ear. tdTom positive fibers, but not cell bodies, were detected in principal sensory nucleus (Pr5) and in the lateral lemniscus (ll). Forebrain and cortical regions were devoid of *Kcnip2*^{GlyT2}-tdTom neurons. Thus, Cre-mediated recombination in *Kcnip2*^{roxCre}*;GlyT2::Dre* mice was found to be mostly restricted to the spinal cord and brainstem, while it was completely absent from DRGs and forebrain areas. Scale bar, 500 µm.

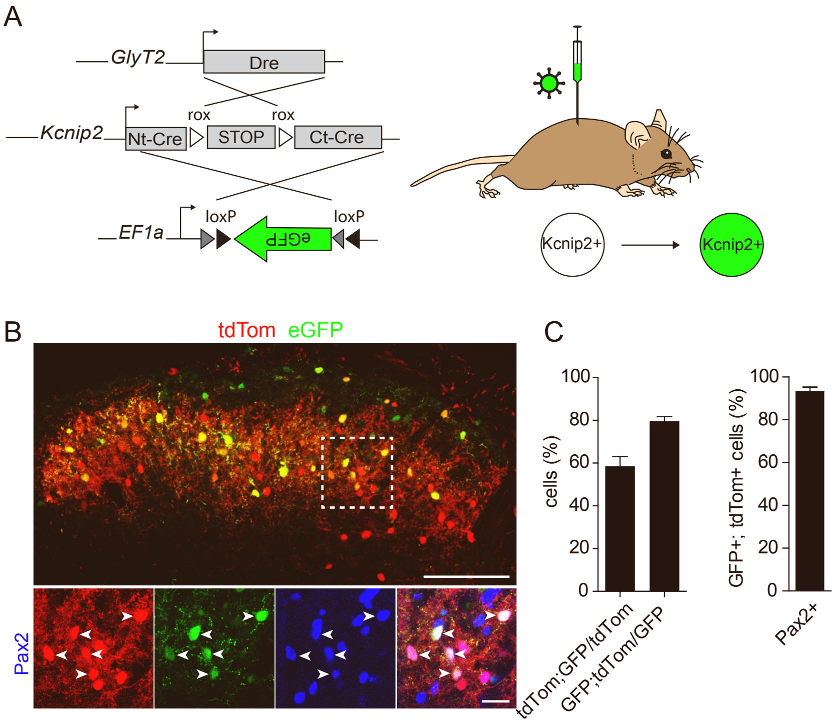

Fig. S6: Efficiency of intersectional AAV-mediated transgene expression in *Kcnip2*^{GlyT2} neurons, Related to Figure 3. A, *Kcnip2*^{GlyT2};*ROSA26*$^{lsl\text{-}tdTom}$ were intraspinally injected with an AAV carrying a cre-dependent eGFP expression cassette. **B**, transverse section of the lumbar spinal cord stained with antibodies against tdTom and eGFP. Scale bars, 100 µm (overview) and 20 µm (high magnification images). **C**, Quantification of co-expression of tdTom with eGFP. n = 12 sections from a total of three mice. Mean ± SEM.

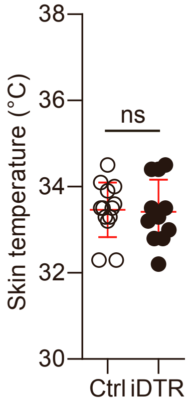

Fig. S7: Skin temperature of *Kcnip2*^{GlyT2} ablated mice, Related to Figure 3. Skin temperature was measured seven days after systemic (intraperitoneal) injection of diphtheria toxin (100 ng/g body weight). n = 11 control mice and n = 9 iDTR mice; ns, not significant (P = 0.88), unpaired t test. Mean ± SEM.

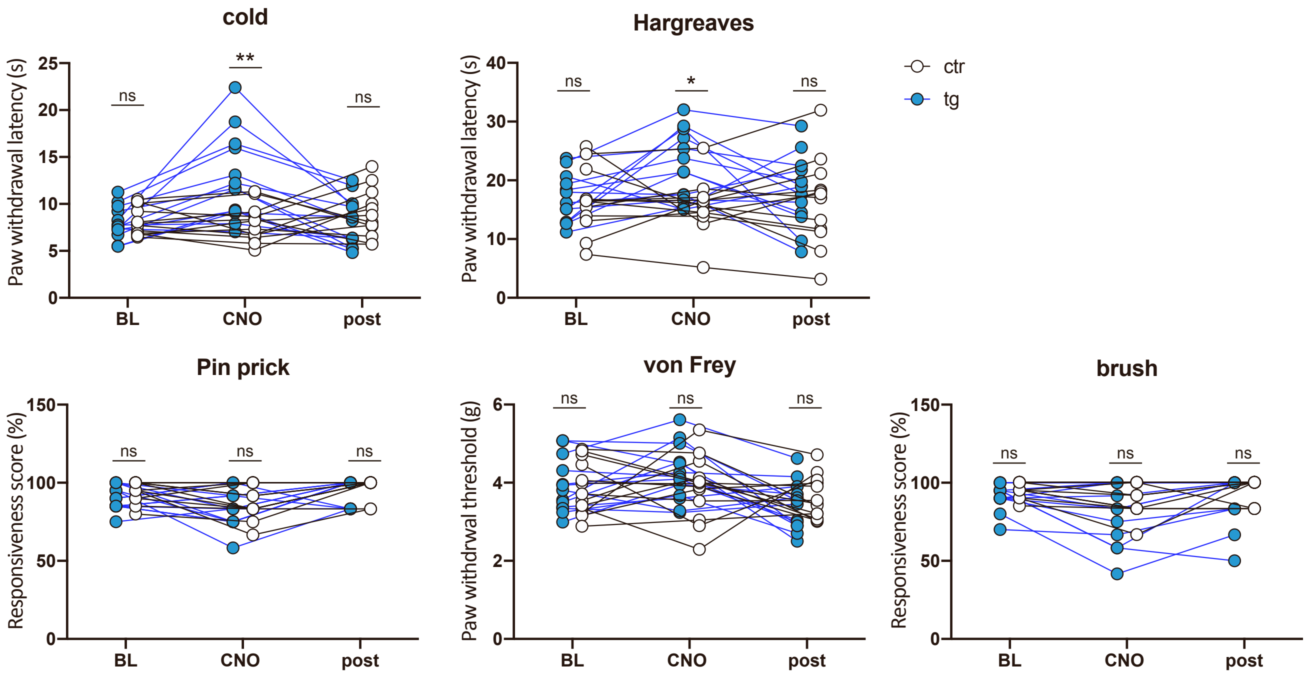

Fig. S8: Time course and reversibility of chemogenetic *Kcnip2*^{GlyT2} neuron activation, Related to Figure 4. Control (ctr) and *Kcnip2*^{GlyT2} (tg) mice were injected with an an AAV carrying a cre-dependent hM3Dq expression cassette. Mice were tested 24 h before CNO injection (BL), 1 – 2 hrs after CNO injection (CNO) and 24 h after CNO injection (post). Two-way ANOVA followed by Bonferroni correction**:** cold F(2,46) = 13.72, P < 0.0001; control versus tg in the presence of CNO: P = 0.0096; Hargreaves F(2,46) = 3.625, P = 0.0345; control versus tg (CNO), P = 0.0100; pin prick F(2,48) = 0.2406, P = 0.7871; control versus tg (CNO): P = 0.6258; von Frey F (2,48) = 1.442, P = 0.2465, control versus tg (CNO), P = 0.6656; brush F(2,48) = 0.8631, P = 0.4283, control versus tg (CNO), P = 0.3641. *, P < 0.05; **, P < 0.01; ns, not significant (P > 0.05). n = 13 for both genotypes.

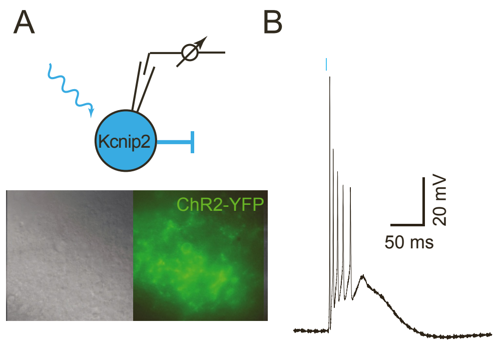

Fig. S9: Optogenetic stimulation of dorsal *Kcnip2*^{GlyT2} neurons, Related to Figure 6. A, Transverse spinal section. Left: IR gradient contrast, right, ChR2-YFP fluorescence. **B**, current clamp recording of a *Kcnip2*^{GlyT2} neuron. Blue line indicates blue light exposure.
