# Supplementary material for: Inhibitory Kcnip2 neurons of the spinal dorsal horn control behavioral sensitivity to environmental cold
Source: Neuron. 2023 Jan 4;111(1):92–105.e5. doi: 10.1016/j.neuron.2022.10.008 (PMC9831669; doi:10.1016/j.neuron.2022.10.008)
Supplement: Document S2. Article plus supplemental information [file mmc2.pdf]

Neuron

Article

Inhibitory *Kcnip2* neurons of the spinal dorsal horn control behavioral sensitivity to environmental cold

Graphical abstract

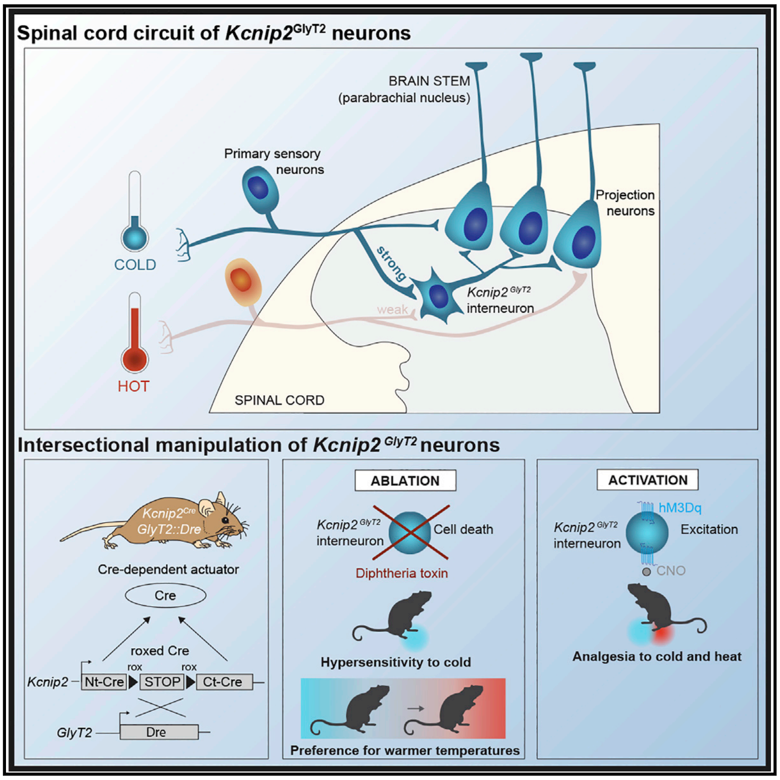

Highlights

- *Kcnip2* defines a population of mainly inhibitory interneurons of the spinal dorsal horn
- *Kcnip2* neurons are activated by cold and heat and inhibit lamina I projection neurons
- Ablation of *Kcnip2* neurons specifically increases behavioral sensitivity to cold
- Activation of *Kcnip2* neurons suppresses cold, heat, and chemical nociception

Authors

Gioele W. Albisetti, Robert P. Ganley, Francesca Pietrafesa, ..., Pawel Pelczar, Hendrik Wildner, Hanns Ulrich Zeilhofer

Correspondence

hwildner@pharma.uzh.ch (H.W.), zeilhofer@pharma.uzh.ch (H.U.Z.)

In brief

Albisetti et al. have developed a new tool (“roxed Cre”) for intersectional targeting of inhibitory *Kcnip2* neurons of the spinal dorsal horn. They show that these neurons are key elements of neural circuits that control sensitivity to environmental cold in mice. The neurons may also contribute to cold-induced analgesia.

Albisetti et al., 2023, Neuron *111*, 92–105
January 4, 2023 © 2022 The Author(s). Published by Elsevier Inc.
https://doi.org/10.1016/j.neuron.2022.10.008

CellPress

CellPress
OPEN ACCESS

Neuron

Article

Inhibitory *Kcnip2* neurons of the spinal dorsal horn control behavioral sensitivity to environmental cold

Gioele W. Albisetti,[1] Robert P. Ganley,[1,7] Francesca Pietrafesa,[1] Karolina Werynska,[1] Marília Magalhaes de Sousa,[1] Rebecca Sipione,[1,8] Louis Scheurer,[1] Michael R. Bösl,[2] Pawel Pelczar,[3] Hendrik Wildner,[1,*] and Hanns Ulrich Zeilhofer[1,4,5,6,9,*]

[1]Institute of Pharmacology and Toxicology, University of Zurich, 8057 Zürich, Switzerland
[2]Institute of Experimental Biomedicine I, University Hospital Würzburg, and Rudolf Virchow Center, University of Würzburg, 97080 Würzburg, Germany
[3]Center for Transgenic Models, University of Basel, 4001 Basel, Switzerland
[4]Institute of Pharmaceutical Sciences, Swiss Federal Institute of Technology (ETH) Zürich, 8093 Zürich, Switzerland
[5]Center for Neuroscience Zurich (ZNZ), 8057 Zürich, Switzerland
[6]Drug Discovery Network Zurich (DDNZ), 8057 Zürich, Switzerland
[7]Present address: National Institute of Dental and Craniofacial Research, National Institutes of Health, Bethesda, MD 20892, USA
[8]Present address: Department of Clinical Neurosciences, University of Geneva, 1211 Geneva, Switzerland
[9]Lead contact
*Correspondence: hwildner@pharma.uzh.ch (H.W.), zeilhofer@pharma.uzh.ch (H.U.Z.)
https://doi.org/10.1016/j.neuron.2022.10.008

SUMMARY

Proper sensing of ambient temperature is of utmost importance for the survival of euthermic animals, including humans. While considerable progress has been made in our understanding of temperature sensors and transduction mechanisms, the higher-order neural circuits processing such information are still only incompletely understood. Using intersectional genetics in combination with circuit tracing and functional neuron manipulation, we identified *Kcnip2*-expressing inhibitory (*Kcnip2*^{GlyT2}) interneurons of the mouse spinal dorsal horn as critical elements of a neural circuit that tunes sensitivity to cold. Diphtheria toxin-mediated ablation of these neurons increased cold sensitivity without affecting responses to other somatosensory modalities, while their chemogenetic activation reduced cold and also heat sensitivity. We also show that *Kcnip2*^{GlyT2} neurons become activated preferentially upon exposure to cold temperatures and subsequently inhibit spinal nociceptive output neurons that project to the lateral parabrachial nucleus. Our results thus identify a hitherto unknown spinal circuit that tunes cold sensitivity.

INTRODUCTION

Proper sensing of environmental cold is essential for organisms to avoid local tissue damage and to prevent potentially life-threatening cooling of the body upon prolonged exposure to below-physiological temperatures. Our body detects a wide range of pleasant, innocuous, and noxious temperatures through a set of dedicated receptors and ion channels, many of which belong to the family of transient receptor potential (TRP) channels. Environmental cold is sensed by TRPM8 channels (McKemy et al., 2002; Peier et al., 2002) and likely by other, potentially still unidentified, channels and receptors that depolarize and excite the peripheral terminals of sensory neurons (Gong et al., 2019; Story et al., 2003; Viana et al., 2002). These neurons subsequently transmit the information to the CNS. As for most other somatosensory modalities, information about cold is first processed in the neural networks of the spinal cord before it is further relayed to the brain by projection neurons. The output of these projection neurons is not a simple function of the sensory input arriving at the spinal cord (e.g., Hachisuka et al., 2018) but is, instead, modulated by local neural networks comprising a plethora of interneurons. In the case of cold, such interneurons fine-tune sensitivity and responses to cold. Furthermore, their activation may potentially contribute to cooling-induced analgesia (Ernst and Fialka, 1994) while their dysfunction may lead to cold allodynia and hyperalgesia, frequent symptoms of chronic neuropathic pain (Jensen and Finnerup, 2014; Maier et al., 2010; for a review see Scadding and Koltzenburg, 2013). Currently, little is known about the identity of interneurons that modulate cold sensitivity.

Work from several groups has set the basis for an unbiased classification of dorsal horn interneurons based on their transcriptomic profile. Although still unproven for many classes, it

 © 2022 The Author(s). Published by Elsevier Inc.
This is an open access article under the CC BY license (http://creativecommons.org/licenses/by/4.0/).

CellPress
OPEN ACCESS

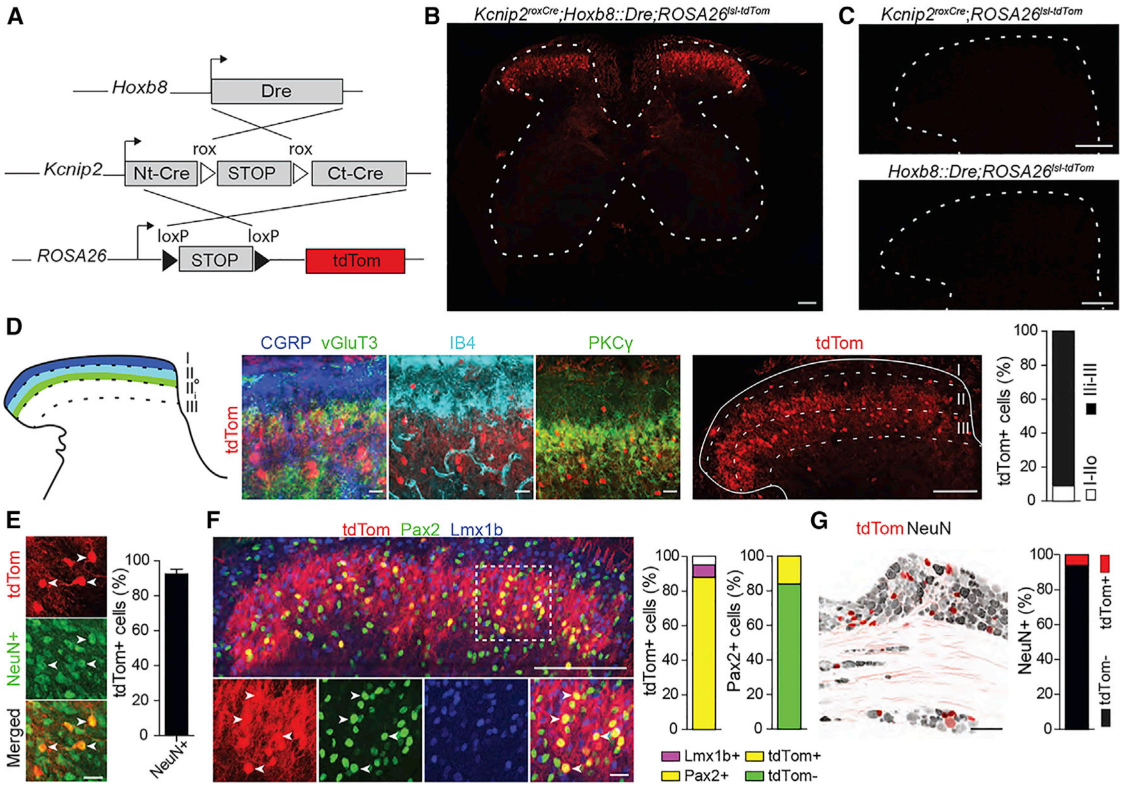

Figure 1. Molecular characterization of spinal *Kcnip2* neurons

(A) Desired recombination events in *Kcnip2*^{roxCre};Hoxb8::Dre;ROSA26$^{lsl\text{-}tdTom}$ mice.

(B) TdTom expression in transverse section of the lumbar spinal cord of *Kcnip2*^{roxCre};Hoxb8::Dre;ROSA26$^{lsl\text{-}tdTom}$ triple transgenic mice.

(C) Same as (B), but in mice lacking either the *Kcnip2*^{roxCre} or Hoxb8::Dre transgene. Scale bar, 100 μm.

(D) *Kcnip2*-tdTom neurons (red) in transverse sections of the lumbar spinal cord relative to lamina-specific markers CGRP (blue), vGluT3 (green), IB4 (cyan), and PKCγ (green). Six sections from two mice. Scale bar, 20 μm.

(E) *Kcnip2* cells co-expressing the neuronal marker NeuN (green). n = 4 sections from three mice. Scale bar, 100 μm. Mean ± SEM.

(F) Co-expression of tdTom (red) with Pax2 (green; inhibitory neurons) and Lmx1b (blue; excitatory neurons). n = 7 sections from two mice; Scale bars: 100 μm (top), 20 μm (bottom). Bar charts: n = 11 sections from three mice.

(G) Lumbar DRG with *Kcnip2* neurons (red) and NeuN staining (gray). n = 6 sections from two mice. Scale bar, 100 μm.

is generally assumed that such genetically defined populations serve specific physiological functions (for a review, see Dobrott et al., 2019). A previous genome-wide transcriptome screen from our group (Wildner et al., 2013) has identified *Kcnip2* as a marker gene of a population of mainly inhibitory interneurons located in dorsal horn laminae II and III. Abraira et al. (2017) have used *Kcnip2* as a marker for interneurons located in the "mechanosensory dorsal horn." *Kcnip2* encodes for a voltage-gated potassium channel interacting protein whose role has been extensively studied in cardiac physiology. Except for its recent identification as a marker of retinal ganglion neurons that are required for the detection of looming objects and subsequent escape and freezing behaviors (Wang et al., 2021), its expression in the CNS has not attracted much attention. To identify physiological functions of *Kcnip2* neurons in sensory processing, we generated a Dre-dependent Cre recombinase transgenic mouse for intersectional targeting of these neurons. Both local and more widespread ablation of inhibitory *Kcnip2* neurons from the mouse spinal dorsal horn identified a critical function of these neurons in the tuning of behavioral sensitivity to environmental cold. Anatomical circuit tracing and combined electrophysiological/optogenetic experiments in spinal cord slices demonstrated that inhibitory dorsal horn *Kcnip2* neurons are placed at a strategic site between cold-sensitive sensory afferents and lamina I spinoparabrachial projection neurons. Finally, chemogenetic activation experiments suggest that they also contribute to cooling-induced analgesia.

RESULTS

Intersectional approach to study *Kcnip2* neurons in the mouse spinal cord

To identify, characterize, and manipulate *Kcnip2* neurons of the spinal dorsal horn or subsets of these neurons, we generated *Kcnip2*^{roxCre} mice, which carry an intersectional Dre-dependent Cre recombinase sequence (Hermann et al., 2014) targeted to the *Kcnip2* locus. Dre-dependence was achieved through the inclusion of a STOP cassette flanked by a pair of rox sites into the Cre sequence (Figures S1A and S1B). In a first experiment, we crossed the *Kcnip2*^{roxCre} mice with *Hoxb8::Dre* mice

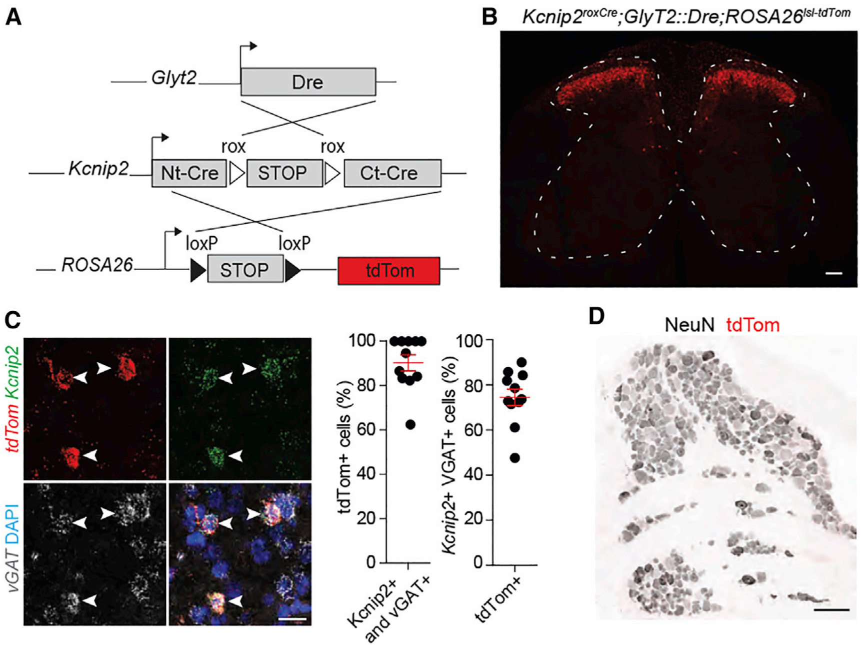

Figure 2. Molecular characterization of *Kcnip2*^{GlyT2} neurons in the spinal cord and DRG

(A) Recombination strategy for *Kcnip2 GlyT2* intersectional targeting. Ct-Cre and Nt-cre denote C- and N-terminal Cre fragments.

(B) Transverse section of lumbar spinal cord of *Kcnip2*^{roxCre};GlyT2::Dre;ROSA26$^{lsl-tdTom}$ triple transgenic mice. Red, *Kcnip2* neurons. Scale bar, 100 μm.

(C) Multicolor *in situ* hybridization. mRNA expression of tdTom (red), *Kcnip2* (green), and vGAT (gray). DAPI (4′,6-diamidino-2-phenylindole) staining shown in blue, n = 11 sections from two mice. Scale bar, 20 μm. Mean ± SEM.

(D) No tdTom neurons were found in lumbar DRGs. Scale bar, 100 μm.

(Figure 1A; for the generation and gross characterization of *Hoxb8::Dre* mice see Figure S2). This approach was chosen to express functional Cre in the whole spinal cord and in peripheral sensory (dorsal root ganglia [DRG]) neurons (compare also Witschi et al., 2010). To verify the fidelity of the roxCre construct, we crossed double transgenic *Kcnip2*^{roxCre}*;Hoxb8::Dre* mice with Cre-dependent tdTom (*ROSA26*$^{lsl-tdTom}$; Ai14) reporter mice. In transverse sections of the lumbar spinal cords prepared from the offspring of these crosses, tdTom positive cells were only detected in triple transgenic *Kcnip2*^{roxCre}*; Hoxb8::Dre;ROSA26*$^{lsl-tdTom}$ (Kcnip2^{Hoxb8}-tdTom) mice (Figure 1B) but not in double transgenic mice that lacked either Cre or Dre (Figure 1C). We then used these *Kcnip2*^{Hoxb8}-tdTom mice for the first morphological characterization of *Kcnip2* neurons in the lumbar spinal cord. To allow unequivocal identification of the different dorsal horn laminae, we stained transverse sections with antisera against calcitonin gene-related peptide (CGRP), vGluT3, and protein kinase Cγ (PKCγ) and with isolectin B4 (IB4), enabling us to identify the boundaries between laminae I, II outer (IIo), II inner (IIi), and III (Figure 1D). tdTom positive somata were located at high density in laminae IIi and III. Some tdTom neurons were also found scattered in laminae I and IIo, while no tdTom neurons were found in the ventral horn. The vast majority (92.6% ± 2.7%) of tdTom somata (a total of 214 in four sections from four mice) were positive for the neuronal marker NeuN (Figure 1E). Co-staining with Lmx1b and Pax2, respective markers of excitatory and inhibitory neurons, revealed that most *Kcnip2* neurons (90.2% ± 1.3% of a total of 405 *Kcnip2*^{Hoxb8}-tdTom neurons in seven sections from two mice) were inhibitory, while only 7.2% ± 0.8% expressed the excitatory marker Lmx1b (Figure 1F). Conversely, *Kcnip2*^{Hoxb8} neurons constituted 16.1% ± 0.5% of the total population of Pax2-positive inhibitory interneurons. Of all *Kcnip2*^{Hoxb8}-tdTom neurons analyzed in nine sections from three mice per marker, 56.8% ± 4.0% co-expressed Gbx1, 39.7% ± 3.8% parvalbumin (PV), 19.1% ± 1.1% cMaf, and 7.4% ± 1.4% PKCγ (Figure S3A). *Kcnip2*^{Hoxb8}-tdTom neurons were not only present in the spinal dorsal horn but also in the DRG, where they accounted for 5.6% ± 0.7% of all neurons (Figure 1G; for a neurochemical characterization of *Kcnip2*-positive DRG neurons, see Figure S3B).

Intersectional genetic strategy targeting inhibitory *Kcnip2* neurons of the spinal cord and hindbrain

We next followed a strategy to selectively manipulate inhibitory *Kcnip2* neurons in the spinal cord and hindbrain without targeting the small excitatory subpopulation of *Kcnip2* spinal neurons or *Kcnip2* DRG neurons. To this end, we used a bacterial artificial chromosome (BAC) transgene containing a Dre coding sequence expressed under the transcriptional control of the GlyT2 (*Slc6a5*) gene, an established marker of glycinergic neurons (Poyatos et al., 1997; Zeilhofer et al., 2005; Figure S4). Because GlyT2 is transiently expressed in all inhibitory neurons of the spinal cord during development (Foster et al., 2015; Zeilhofer et al., 2005), the *GlyT2::Dre* mouse allows recombination not only in glycinergic inhibitory neurons but also in inhibitory neurons that become purely GABAergic in adulthood. Of note, GlyT2::Dre recombination spares the forebrain where GlyT2 is never expressed. We crossed *Kcnip2*^{roxCre}*;GlyT2::Dre* mice with *ROSA26*$^{lsl-tdTom}$ reporter mice to obtain triple transgenic *Kcnip2*^{GlyT2}-tdTom mice (Figure 2A). In these mice, tdTom neurons were again detected in the spinal dorsal horn (Figure 2B). The vast majority of *Kcnip2*^{GlyT2}-tdTom neurons co-expressed mRNAs encoding *Kcnip2*, *tdTom* and *vGAT*, verifying their inhibitory phenotype and the eutrophic expression of cre (Figure 2C). No tdTom neurons were found in DRG (Figure 2D). Outside the spinal cord, *Kcnip2*^{GlyT2}-tdTom neurons were present in several brainstem regions, including nuclei of the somatosensory and auditory systems but not in the forebrain (Figure S5).

Reduced sensitivity to environmental cold in *Kcnip2*^{GlyT2} neuron-ablated mice

To gain insights into the potential functions of dorsal horn *Kcnip2*^{GlyT2} neurons, we turned to adeno-associated virus (AAV)-mediated gene transfer for ablation and functional manipulation experiments. Before starting these behavioral experiments, we investigated the efficacy of intersectional AAV-mediated transgene expression in *Kcnip2*^{GlyT2} neurons. To this end, we compared the Cre-dependent expression of a viral transgene (eGFP) with the expression of tdTom originating from the *ROSA26*$^{lsl-tdTom}$ reporter (Figure S6). In the lumbar segments targeted by AAV injection, 60.0% ± 7.7% (five sections from two mice) of the tdTom neurons also expressed eGFP. Vice versa, 79.7% ± 2.0% of eGFP-positive neurons expressed tdTom. The vast majority of eGFP;tdTom neurons (93.1% ± 2.2%) also expressed Pax2, confirming their inhibitory nature.

We then tested whether local diphtheria toxin (DTX)-mediated ablation of *Kcnip2*^{GlyT2} neurons would interfere with behavioral responses to different somatosensory and nociceptive stimuli. *Kcnip2*^{GlyT2} neurons were virally transduced with the DTX A (DTA) subunit, which inhibits protein synthesis and subsequently induces cell death. We injected AAVs (AAV1.EF1a.flex.DTA) carrying a Cre-dependent DTA transgene into the left lumbar spinal cord of *Kcnip2*^{GlyT2};ROSA26$^{lsl-tdTom}$ mice (Figure 3A). Littermates of *Kcnip2*^{GlyT2} mice that lacked the GlyT2::Dre and/or the *Kcnip2*^{roxCre} transgene but had undergone the same virus injections were used as controls. The *ROSA26*$^{lsl-tdTom}$ allele was included to confirm the local loss of *Kcnip2*^{GlyT2} neurons after virus injection (Figure 3B). Three to four weeks after unilateral AAV injection into the left lumbar spinal cord segments L3–L5, mice underwent a battery of sensory tests applied to the left hind paw (Figure 3C). Local ablation of spinal *Kcnip2*^{GlyT2} neurons markedly increased cold sensitivity with paw withdrawal latencies of 8.0 ± 0.8 s in *Kcnip2*^{GlyT2} ablated mice (n = 10) versus 14.1 ± 1.5 s in nonablated control mice (n = 12) (p = 0.003, unpaired t test). By contrast, *Kcnip2*^{GlyT2} ablated mice did not show altered responses to noxious radiant heat stimulation (response latencies: 22 ± 1.5 s versus 20.9 ± 1.2 s, p = 0.59), noxious mechanical (pin-prick) stimulation (response scores: 84.2% ± 6.1% versus 96% ± 3.1%, p = 0.12), punctate mechanical stimulation with von Frey filaments (response thresholds: 4.6 ± 0.2 g versus 4.1 ± 0.2 g, p = 0.1), or innocuous dynamic mechanical brush stimulation (response scores: 76.7% ± 7.6% versus 94.0% ± 4.0%). No change in rotarod performance was observed (latencies to fall: 121.7 ± 14.2 s versus 134.0 ± 22.2 s, p = 0.63).

We next tested whether ablation of *Kcnip2*^{GlyT2} neurons would also result in an altered temperature preference in the thermal gradient test (Shimizu et al., 2005). As the local unilateral ablation strategy employed in the sensory tests above would likely not produce meaningful results in this experiment, we aimed for more widespread ablation of *Kcnip2*^{GlyT2} neurons from the whole spinal cord. To this end, we used systemic (intraperitoneal [i.p.]) injections of DTX (0.1 mg/kg body weight) in triple transgenic *Kcnip2*^{roxCre}; *GlyT2::Dre;ROSA26*$^{lsl-iDTR}$ (*Kcnip2*^{GlyT2}-iDTR) mice. In these mice, a Cre-dependent DTX receptor (iDTR transgene; Buch et al., 2005) is expressed in all *Kcnip2*^{GlyT2} neurons. Systemic DTX injection should therefore ablate iDTR-expressing *Kcnip2*^{GlyT2} neurons throughout the spinal cord (Figure 3D). Littermates of *Kcnip2*^{GlyT2}-iDTR mice lacking the Cre and/or Dre transgenes served as controls. We first verified the efficacy of this ablation strategy and found that a single injection of DTX reduced the number of tdTom labeled *Kcnip2*^{GlyT2} neurons in the spinal cord by about half, from 49.6 ± 1.8 cells per section in control mice to 22.3 ± 2.4 in triple transgenic mice ($p = 3 \times 10^{-7}$, 9 sections from three mice per group) (Figure 3E). We then continued with the behavioral experiments and compared temperature preferences in a rectangular temperature gradient 13°C–39°C apparatus (Figure 3F). The position and movement of the mice were continuously video-recorded for 30 min immediately after placing the mice into the thermal gradient apparatus. During these 30 min, mice exhibited intense exploratory behavior. Control mice did not display an obvious temperature preference in the range between 15°C and 33°C. They spent more time in the most extreme cold and warm temperatures, presumably due to a preference for the end-corners of the elongated gradient chamber. By contrast, *Kcnip2*^{GlyT2} ablated mice showed a clear aversion to cold temperatures. Control mice spent 521 ± 64 s in the colder area (13°C–22°C) compared with 227 ± 27 s in *Kcnip2*^{GlyT2} ablated mice (p = 0.002, n = 7 and 10 for control and *Kcnip2*^{GlyT2} ablated mice). This change was accompanied by a presumably compensatory increase in time spent in the warmer temperature zone (28°C–39°C) (606 ± 67 s in control versus 1,006 ± 62 s in *Kcnip2*^{GlyT2} ablated mice; p = 0.0008). The median preferred temperature control and of *Kcnip2*^{GlyT2} neuron-ablated mice and control mice differed by more than 4°C (24.8°C ± 1.2°C versus 29.1°C ± 0.2°C for control and *Kcnip2*^{GlyT2} ablated mice, respectively, p = 0.01). Of note, no difference was found in the skin temperature between *Kcnip2*^{GlyT2} and control mice (Figure S7).

Chemogenetic activation of spinal *Kcnip2*^{GlyT2} neurons

In order to gain further insight into the contribution of *Kcnip2*^{GlyT2} neurons to the regulation of cold sensitivity, we employed a chemogenetic activation strategy and injected the left lumbar spinal cord segments L3–L5 of *Kcnip2*^{roxCre};*GlyT2::Dre* mice with an AAV carrying a Cre-dependent hM3Dq transgene (AAV1.hSyn.flex.hM3Dq) (Figure 4A). Mice lacking the *GlyT2::Dre* and/or the *Kcnip2*^{roxCre} transgene served again as controls. Expression of hM3Dq was visualized through mCherry fluorescence (Figure 4B), which was fused in-frame to the hM3Dq coding sequence. We then applied the same sensory test battery as previously used in mice after *Kcnip2*^{GlyT2} neuron ablation (Figures 4C and S8). All mice were injected with clozapine-N-oxide (CNO, 2 mg/kg, i.p.) 1 h before the start of the sensory testing. Chemogenetic activation of *Kcnip2*^{GlyT2} neurons significantly increased response latencies in the cold plantar test from 8.2 ± 0.6 s in *Kcnip2*^{GlyT2}-hM3Dq mice to 12.7 ± 1.3 s in control mice (p = 0.004, unpaired t test, n = 13 for both groups). While ablation of *Kcnip2*^{GlyT2} neurons had no effect on heat sensitivity, chemogenetic activation of *Kcnip2*^{GlyT2} neurons caused a statistically significant decrease in noxious heat sensitivity (withdrawal latencies: 16.8 ± 1.7 s versus 22.5 ± 1.6 s; p = 0.024). Responses to noxious or innocuous mechanical stimulation showed a trend toward reduced sensitivity that did not reach statistical significance (pin-prick response score: 89% ± 3% versus 83% ± 3%, for control versus hM3Dq mice, p = 0.21 unpaired t test; von Frey test

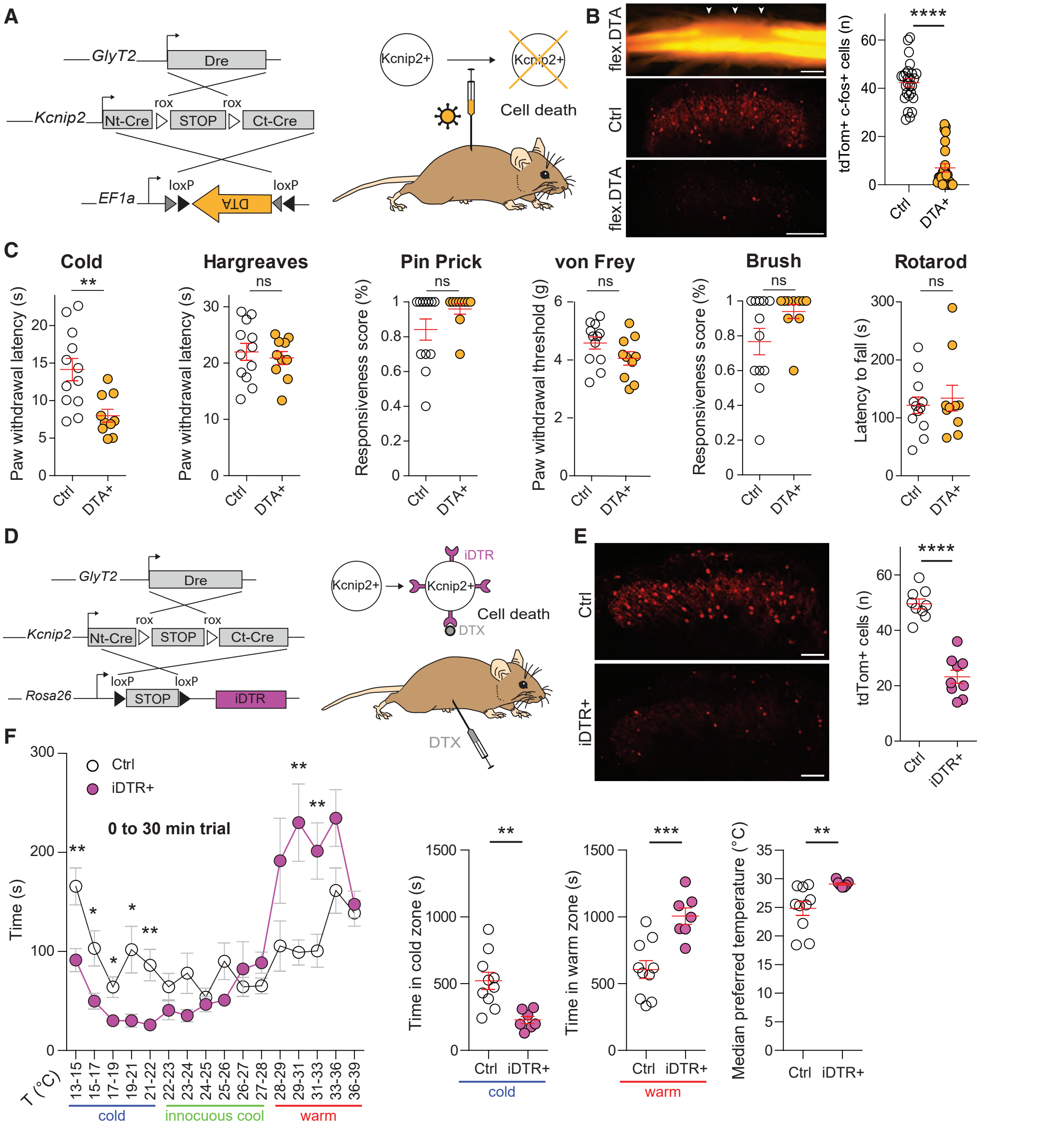

Figure 3. DTX-mediated ablation of *Kcnip2*^{GlyT2} neurons

(A–C) Local ablation of *Kcnip2*^{GlyT2} neurons using AAV1.Ef1a.flex.DTA.

(A) Experimental strategy. Unilateral AAV injections were made into the lumbar dorsal horn of *Kcnip2*^{roxCre};GlyT2::Dre;ROSA26$^{lsl-tdTom}$ triple transgenic mice.

(B) (Left, top) Whole-mount spinal cord after *Kcnip2*^{GlyT2} ablation. White arrows indicate injection sites. Scale bar, 1 mm. (Middle and bottom) Loss of *Kcnip2*^{GlyT2} neurons (red) in the dorsal horn of AAV1.Ef1a.flex.DTA injected mice. Scale bar, 100 μm. (Right) Quantification of DTA mediated loss of *Kcnip2*^{GlyT2} neurons. n = 25 sections from five mice per group.

(C) Nociceptive sensitivity and motor performance of *Kcnip2*^{GlyT2} neuron-ablated mice, 3–4 weeks after AAV injection. n = 10 *Kcnip2*^{GlyT2} ablated mice, and n = 12 control mice. Scale bar, 1 mm.

(D–F) Spinal cord-wide ablation of *Kcnip2*^{GlyT2} neurons.

(D) Experimental strategy, i.p. injection of complete diphtheria toxin (DTX) in *Kcnip2*^{roxCre}; GlyT2::Dre;ROSA26$^{lsl-iDTR}$ triple transgenic mice.

(E) Loss of tdTom (*Kcnip2*^{GlyT2}) neurons in the dorsal horn of DTX treated mice. n = 9 sections from three mice per group; Scale bar, 100 μm.

(F) Thermal gradient test. (Left) Time spent by *Kcnip2*^{GlyT2} ablated and control mice in different temperature zones during a 30-min interval. (Right) Quantification of time spent in cold (13°C–22°C) and warm (28°C–39°C) zones. Median preferred temperature in *Kcnip2*^{GlyT2} ablated (n = 7) and control mice (n = 10); *p < 0.05, **p < 0.01, ***p < 0.001, ****p < 0.0001; unpaired t test. Mean ± SEM.

CellPress
OPEN ACCESS

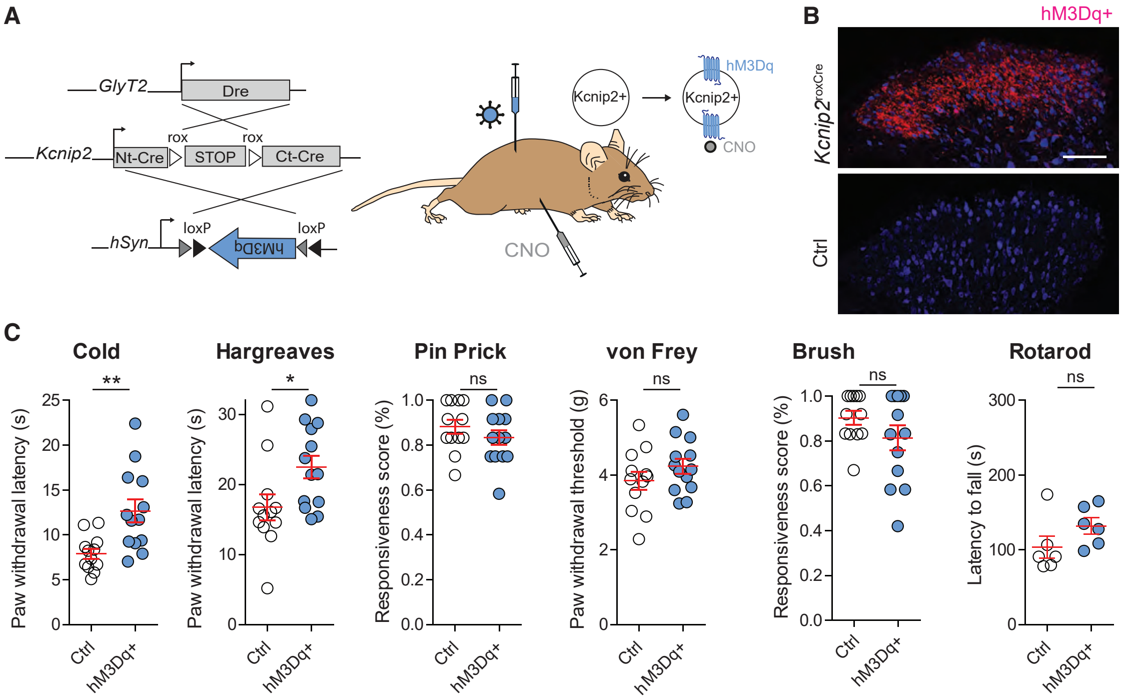

Figure 4. Chemogenetic activation of *Kcnip2*^{GlyT2} neurons

(A) Experimental strategy. AAVs carrying a Cre-dependent hM3Dq-mCherry expression cassette (AAV1.hsyn.flex.hM3dq.mCherry) were injected unilaterally into the lumbar spinal cord tissue of *Kcnip2*^{roxCre};GlyT2::Dre mice.

(B) Expression of hM3Dq-mCherry 2 weeks after AAV injection. Scale bar, 100 μm.

(C) Behavioral tests. CNO was administered systemically (i.p.) 2 weeks after AAV injection. Measurements taken between 1 and 3 h after CNO injection. n = 13 mice per group for all sensory tests, n = 6 per group in the rotarod test. Mean ± SEM. *p < 0.05; **p < 0.01; ns, not significant (p > 0.05). For the time course and reversibility of the CNO effect see Figure S8. Note that the sensory testing after chemogenetic activation was done in 7-week-old mice, whereas mice after ablation (Figure 3) were tested at 9 weeks of age. This age difference likely underlies the different baseline latencies in the cold plantar test in both experiments.

threshold: 3.9 ± 0.2 g versus 4.2 ± 0.2 g, p = 0.22; brush test response score: 91% ± 3% versus 81% ± 5%, p = 0.13). Rotarod performance was also unaltered (latencies to fall of 103.3 ± 14.7 s versus 132.0 ± 10.7 s; in control versus *Kcnip2*^{GlyT2}-hM3Dq transgenic mice; p = 0.15). Taken together, the results of the ablation and chemogenetic activation experiments indicate a critical role of *Kcnip2*^{GlyT2} neurons in spinal cold processing.

Integration of *Kcnip2*^{GlyT2} neurons in a dorsal horn circuit for tuning cold sensations

What is the underlying circuit? We first tested whether lumbar dorsal horn *Kcnip2*^{GlyT2}-tdTom neurons became activated by cold stimulation. Exposure of the left hind paw to noxious cold (−20°C acetone) induced widespread c-fos expression determined 2 h after stimulation. Increases in c-fos expression were found when all dorsal horn neurons were analyzed and when only the *Kcnip2*^{GlyT2} population was analyzed (Figure 5A). We then asked if *Kcnip2*^{GlyT2} neurons were particularly sensitive to activation by cold. To this end, we compared the cold-induced increase in c-fos expression in *Kcnip2*^{GlyT2} neurons with the increases evoked by heat (52°C water) and mechanical force (forceps pinch) (Figures 5B and 5C). As expected, all three stimuli increased the total number of c-fos-positive neurons in the ipsilateral dorsal horn. Restricting the analyses to *Kcnip2*^{GlyT2}-tdTom neurons revealed that cold exposure increased the percentage of c-fos-positive *Kcnip2*^{GlyT2} neurons by a factor of 3.4 (from 6.7% ± 2.3% to 22.8% ± 3.9%; p ≤ 0.01, unpaired t test), while heat, which also had a significant effect, only led to an increase by a factor of 1.8 (from 10.4% ± 2.3% to 19.1% ± 3.2%; p ≤ 0.05). Noxious pinch failed to significantly activate *Kcnip2*^{GlyT2} neurons. These data suggest that noxious cold activates *Kcnip2*^{GlyT2} neurons more efficiently than other sensory modalities.

We then used targeted whole-cell recordings of *Kcnip2*^{GlyT2} neurons in transverse slices of the lumbar spinal cord to investigate synaptic input onto and output from *Kcnip2*^{GlyT2} neurons. We first verified the presence of excitatory input from cold-sensing fibers onto *Kcnip2*^{GlyT2} neurons and then monitored changes in the frequency of excitatory postsynaptic currents (EPSCs) upon exposure to icilin (20 μM), a potent activator of TRPM8 channels (Behrendt et al., 2004). Icilin significantly increased the frequency of spontaneous EPSCs in *Kcnip2*^{GlyT2} neurons of laminae I-IIo (Figures 6A and 6B; Kolmogorov-Smirnov test, p < 0.001, n = 7 neurons), but not in neurons located more deeply in laminae IIi and III (p = 0.23, n = 6 neurons), consistent with the termination area of TRPM8-expressing fibers in the superficial dorsal horn (Dhaka et al., 2008).

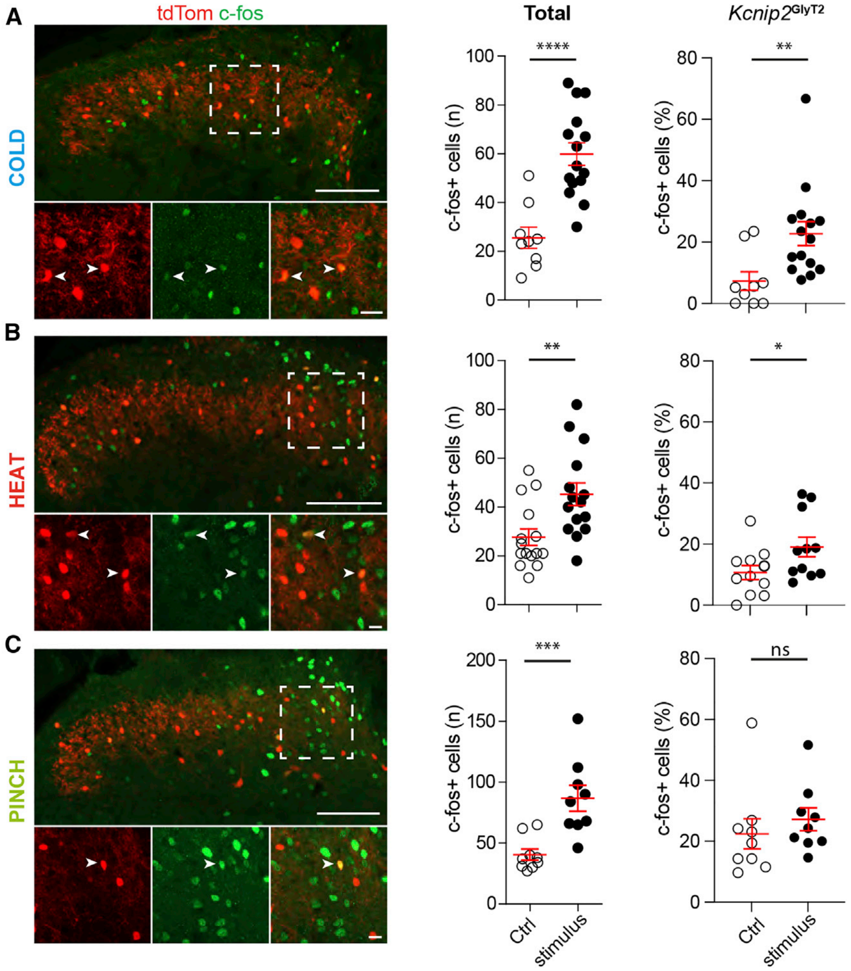

Figure 5. Activation of *Kcnip2*^{GlyT2} neurons by different noxious stimuli

(A) (Left) c-fos-expressing neurons in the lumbar dorsal horn of *Kcnip2*^{GlyT2}-tdTom mice 2 h after cold exposure (−20°C acetone) of the left hind paw. Lower panels higher magnifications of the area indicated above. Arrow heads indicate c-fos-positive *Kcnip2*^{GlyT2}-tdTom neurons. Scale bar, 100 μm overviews and 20 μm insets. (Middle) Total numbers of c-fos-expressing neurons per section. Control, n = 9 sections from three mice; stimulation, n = 14 sections from four mice. (Right) Percentage of *Kcnip2*^{GlyT2}-tdTom neurons expressing c-fos. Control, anesthetized mice without paw stimulation.

(B and C) Same as (A), but exposure to noxious heat (52°C water) (B), or noxious pinch stimulation (C).

Mean ± SEM. *p < 0.05; **p < 0.01; ***p < 0.001, ****p < 0.0001, ns, not significant (p > 0.05). n = 11 sections from three mice (B) and n = 9 sections from three mice (C).

Next, we addressed the postsynaptic targets of spinal *Kcnip2*^{GlyT2} neurons and injected *Kcnip2*^{GlyT2}-tdTom mice with AAVs carrying a Cre-dependent transgene encoding the transsynaptic tracer wheat germ agglutinin (WGA). To focus our analyses on monosynaptic connections, spinal cord tissue was harvested on day 7 after virus injection (Braz et al., 2002). Primary infected *Kcnip2*^{GlyT2} neurons were identified by the co-expression of tdTom and WGA immunostaining, while WGA-positive neurons with no tdTom expression were considered as neurons postsynaptic to *Kcnip2*^{GlyT2} neurons. The majority of postsynaptic neurons were located in laminae I and II (78.8% ± 2.3%), while the rest (21.2% ± 2.3%) were located in lamina III and deeper (Figure 6C). About half of the postsynaptic neurons (55.5% ± 2.8% of a total of 396) expressed the excitatory marker Lmx1b, while the other half (49.1% ± 3.7%) were Pax2 positive (Figure 6D). Closer inspection revealed that most postsynaptic neurons were found in the same laminae as the majority of *Kcnip2*^{GlyT2} neurons, but a few were present in lamina I, which harbors spinal nociceptive output neurons, including those that convey information about cold (Hachisuka et al., 2020). As WGA labeled neurons in lamina I may include nociceptive output neurons, we tested whether *Kcnip2*^{GlyT2} neurons would directly inhibit lamina I output neurons. We used an AAV2retro carrying a tdTom transgene to label lamina I projection neurons from the lateral parabrachial nucleus (*lPbN*), a well-established projection area of dorsal horn nociceptive output neurons. We combined targeted whole-cell recordings from the retrogradely labeled lamina I output neurons with optogenetic stimulation of dorsal horn *Kcnip2*^{GlyT2} neurons. We first verified the efficacy of optogenetic stimulation in triple transgenic *Kcnip2*^{roxCre}*;GlyT2::Dre; ROSA26*$^{lsl\text{-}ChR2}$ (*Kcnip2*^{GlyT2}-ChR2) mice. Wide-field illumination of spinal cord slices with brief 4 ms blue (473 nm) light pulses induced short bursts of usually 3–4 action potentials in *Kcnip2*^{GlyT2} neurons (Figure S9). We then recorded light-evoked inhibitory postsynaptic currents (IPSCs) from retrogradely labeled lamina I projection neurons by stimulating *Kcnip2*^{GlyT2} neurons (Figures 6E and 6F). IPSCs were found in six of seven retrogradely labeled neurons and had an average amplitude of −599 ± 315 pA (n = 6) and were completely blocked by a combination of the $GABA_A$ and glycine receptor blockers bicuculline (20 μM) and strychnine (0.5 μM). Following optogenetic stimulation, the first light-evoked IPSCs occurred with an average latency of 13.7 ± 1.6 ms and had a jitter of 2.0 ± 0.5 ms. Synaptic failures (absence of a postsynaptic response after light stimulation) were observed only in one of six neurons suggesting the presence of monosynaptic connections between *Kcnip2*^{GlyT2} neurons and lamina I projection neurons. In line with previous data by Abraira et al. (2017), these results indicate that *Kcnip2*^{GlyT2} neurons are elements of a feedforward circuit, which inhibits a rather broad range of lamina I output neurons.

The latter finding opens the possibility that *Kcnip2*^{GlyT2} neurons contribute to cooling-induced analgesia, a well-known phenomenon present in rodents (Kanui, 1985; Knowlton et al., 2013)

CellPress
OPEN ACCESS

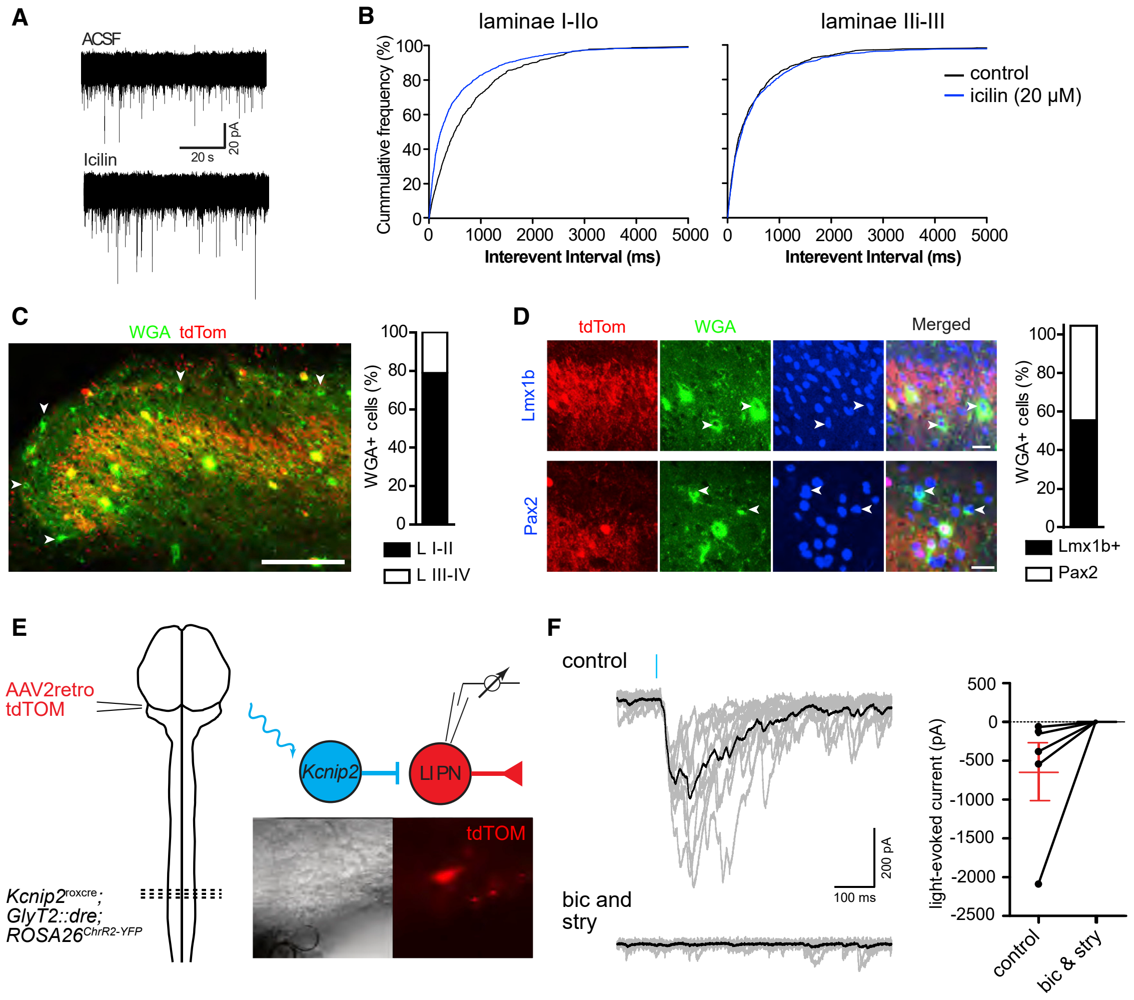

Figure 6. Presynaptic input to and postsynaptic targets of dorsal horn *Kcnip2*^{GlyT2} neurons

(A) Spontaneous EPSCs in a *Kcnip2*^{GlyT2}-tdTom neuron in the absence and presence of 20 μM icilin.

(B) Cumulative frequency distribution of EPSC interevent intervals.

(C) Anterograde tracing from *Kcnip2*^{GlyT2} neurons. Green (WGA)-only neurons are postsynaptic targets of *Kcnip2*^{GlyT2}-tdTom neurons (red). Scale bar, 100 μm.

(D) Excitatory (Lmx1b) or inhibitory (Pax2) neurons postsynaptic to *Kcnip2*^{GlyT2}-tdTom neurons.

(E) Retrograde labeling of lamina I spinoparabrachial neurons with tdTom and optogenetic stimulation of *Kcnip2*^{GlyT2} neurons.

(F) (Left) Representative IPSC traces of a lamina I spinoparabrachial neuron evoked by optogenetic stimulation of *Kcnip2*^{GlyT2} neurons. IPSCs were completely blocked by bicuculline (20 μM) and strychnine (0.5 μM). (Right) Group data, n = 6. Mean ± SEM.

and humans (Bini et al., 1984; Gammon and Starr, 1941). We have tested this hypothesis in the mouse formalin test, an established model of chemically induced pain with known sensitivity to cooling-induced analgesia (Hole and Tjølsen, 1993; Rosland, 1991). Chemogenetic activation of *Kcnip2*^{GlyT2} neurons significantly reduced nocifensive reactions (licking of the injected paw) (p = 0.024, n = 11 and 13, for hM3Dq and control mice, respectively) (Figures 7A and 7B), in agreement with previous results obtained with TRPM8-deficient mice in the same test (Dhaka et al., 2007). Furthermore, we found that chemogenetic activation of *Kcnip2*^{GlyT2} neurons reduces cold allodynia in mice with a chronic constriction injury (CCI) of the sciatic nerve (Figure 7C). The number of nociceptive responses evoked by acetone application to the neuropathic paw was significantly reduced at 1 and 2 h post-CNO injection (18.8 ± 2.79 post CCI versus 9.5 ± 2.27 at 1 h post CNO, and 11.4 ± 3.08 at 2 h post CNO, one-way ANOVA followed by Dunnett's post hoc test p = 0.0007, for 1 h post CNO, p = 0.0075 for 2 h post-CNO). CNO injection did not reduce mechanical hypersensitivity (Figure 7D). These results suggest that spinal *Kcnip2*^{GlyT2} neurons may contribute to cooling-induced analgesia. We next tested whether ablation of *Kcnip2*^{GlyT2} neurons would suppress cooling-induced analgesia in mice with an inflamed hindpaw (Figure 7E). To avoid effects of physical cooling on peripheral nerve conductance (Lowitzsch et al., 1977), we used the TRPM8 agonist menthol (100 mg/kg i.p.; McKemy et al., 2002; Pan et al., 2012; Peier et al., 2002). As expected, ablation of *Kcnip2*^{GlyT2} neurons had no impact on heat-evoked withdrawal

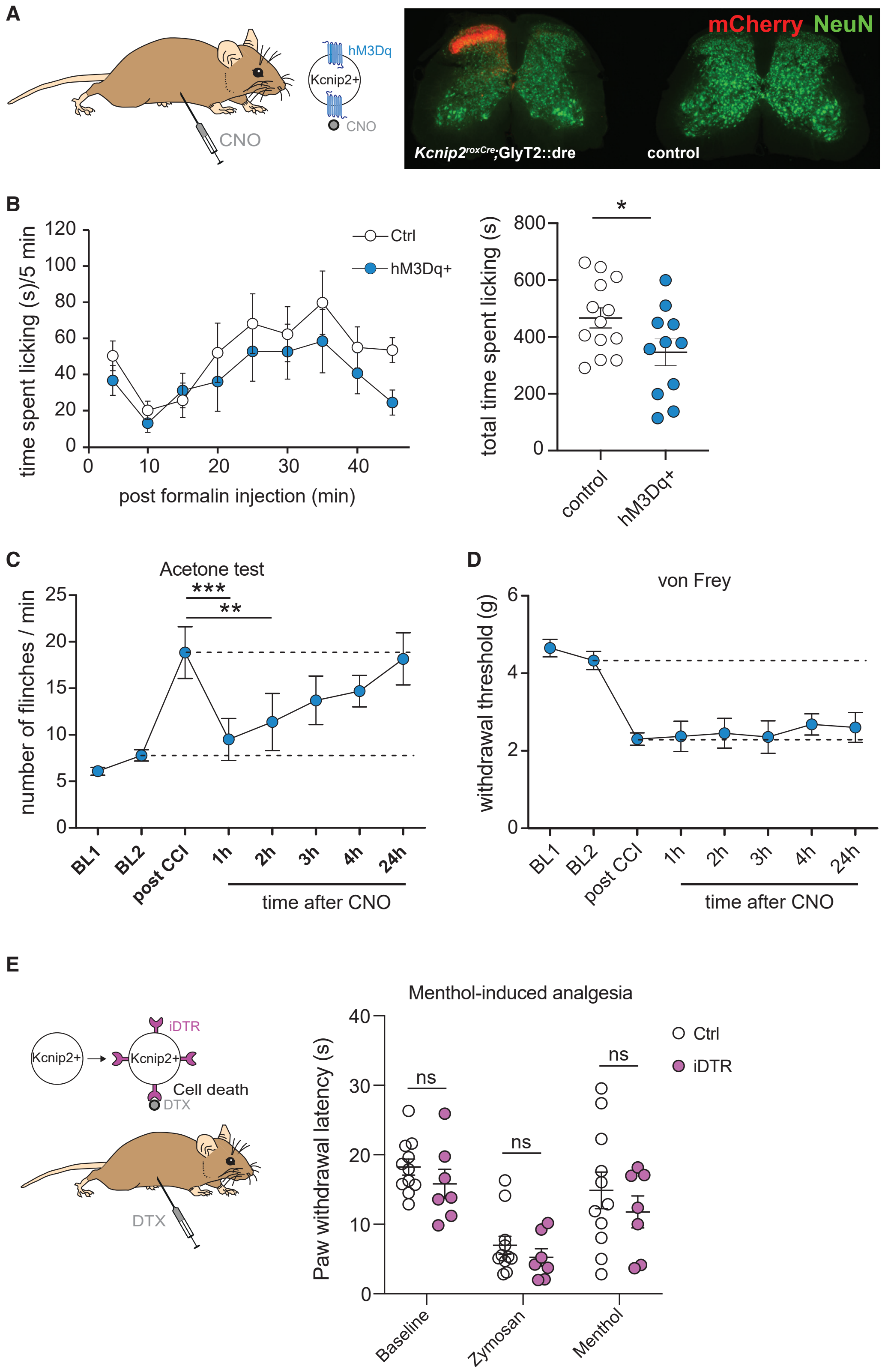

(legend on next page)

CellPress
OPEN ACCESS

latencies at baseline or after injection of zymosan A. However, it also had no significant effect on menthol-induced analgesia. Although unexpected, this result is in line with our electrophysiological data that suggested that TRPM8-positive fibers are not the main source of cooling-induced activation of *Kcnip2*^{GlyT2} neurons.

DISCUSSION

Unbiased classifications of interneurons based on single-cell transcriptome profiles have been established in recent years for many CNS areas. In the spinal cord, three reports have used these techniques and identified depending on the sequencing technique and stringency of clustering between 21 and 43 excitatory and inhibitory neuron classes (Häring et al., 2018; Sathyamurthy et al., 2018; Zeisel et al., 2018). Although an association of specific physiological functions with such neuron classes is highly plausible, obtaining direct evidence has proven very challenging because, in most cases, combinations of several marker genes and recombinases would be required for specific targeting. Here, we have investigated dorsal horn inhibitory interneurons expressing the marker gene *Kcnip2*, which we have previously discovered in a search for nonoverlapping populations of inhibitory dorsal horn neurons (Wildner et al., 2013). These *Kcnip2*^{GlyT2} neurons correspond to the inhibitory neuron classes Gaba14 and Gaba15 of the Ernfors classification (Häring et al., 2018). Consistent with their proposed role as modulators of cold sensitivity, the Ernfors lab found that neurons of the Gaba14 and Gaba15 classes become activated *in vivo* upon stimulation with cold. Our results thus attribute to these neurons, or to subsets of these neurons, a previously unknown function.

Specifically addressing the inhibitory population of dorsal horn *Kcnip2* neurons was only possible using an intersectional targeting strategy. Different tools for such intersectional strategies have been developed. Many approaches depend on reporter constructs that contain two transcriptional STOP signals flanked by recognition sites for different recombinases (such as Cre, Flp, or Dre). Here, we have used an alternative strategy that involves a recombinase (in our case, Cre), whose expression is itself dependent on another recombinase (in our case, Dre). This approach avoids the necessity of creating new double-dependent reporter constructs and instead permits the use of the extensive existing armamentarium of Cre-dependent reporter viruses and mice. Although previous work (Fenno et al., 2014) has raised some concerns about the cross-reactivity of the Cre recombinase with rox sites (the targets of the Dre recombinase), our results show that the system, which depends on the sequential recombination first by Dre and then Cre (Hermann et al., 2014), is highly specific with no signs of recombinase cross-reactivity.

Because GlyT2 is absent from all excitatory neurons, from neurons in the forebrain and from peripheral sensory (DRG) neurons, the intersection of *GlyT2::Dre* and *Kcnip2*^{roxCre} allowed us to specifically target inhibitory *Kcnip2* neurons in the spinal cord and brainstem. We used this approach in combination with Cre-dependent reporter mice and reporter viruses for ablation and chemogenetic and optogenetic activation. Segmental ablation of *Kcnip2*^{GlyT2} neurons reduced cold-induced withdrawal responses with no alternations in responses to heat or mechanical stimuli. More widespread ablation throughout the spinal cord induced a shift in the preferred temperature to higher values. These findings led us to suggest a specific role of dorsal horn *Kcnip2*^{GlyT2} neurons in the spinal control of sensitivity to environmental cold.

Two findings seemingly challenge a specific function of *Kcnip2*^{GlyT2} neurons in controlling cold sensitivity. First, while *Kcnip2*^{GlyT2} neuron ablation exclusively impacted responses to cold, chemogenetic activation reduced both cold and heat responses. Here, it should be considered that results from inhibition or ablation experiments are usually more informative in terms of physiological function than experiments employing neuronal activation because the latter bypass physiological activation and can produce activity patterns not naturally occurring *in vivo*. Hence, if the cold specificity observed in the ablation experiments occurred due to preferential activation of *Kcnip2*^{GlyT2} neurons by cold, then the observed reduction in heat sensitivity following activation of the same neurons would occur because *Kcnip2*^{GlyT2} neurons were activated independently of their sensory input from cold fibers. In support of this concept, we found that cold increased c-fos expression in *Kcnip2*^{GlyT2} neurons more efficiently than heat. A second finding potentially challenging cold specificity was seen in the thermal gradient experiment. Ablation of *Kcnip2*^{GlyT2} neurons throughout the spinal cord not only reduced the time spent in cold areas but also increased the time spent in the warm. Although this increase is likely a consequence of cold avoidance, the warm preference could in principle also originate from a reduction in heat sensitivity. However, if removal of these neurons did reduce heat avoidance in the Hargreaves test, activation, as opposed to ablation, would be expected to produce shorter rather than the observed longer withdrawal latencies. Despite these considerations, heat clearly activates spinal *Kcnip2*^{GlyT2} neurons; yet, their ablation had no effect on heat-evoked withdrawal

Figure 7. Contribution of dorsal horn *Kcnip2*^{GlyT2} neurons to cooling-induced analgesia

(A and B) Formalin test. (A) Left, experimental design. hM3Dq was expressed from AAV1.hsyn.flex.hM3Dq.mCherry in *Kcnip2*^{GlyT2} neurons of the left lumbar dorsal horn. CNO (2 mg/kg, i.p.) was injected 1 h before formalin injection (5%, 20 μl). Right, expression of mCherry tagged hM3Dq (red) 10 days after AAV injection. (B) Time spent licking the injected paw per 5-min intervals (left) and total time spent licking (right) during 45 min post formalin injection. n = 11 and 13, for *Kcnip2*^{GlyT2} transgenic and control mice.

(C and D) CCI-induced cold (C) and mechanical (D) allodynia. Chemogenetic *Kcnip2*^{GlyT2} neuron activation alleviated cold but not mechanical allodynia. n = 6 for both experiments, ***$p < 0.001$, **$p = 0.01$, one-way ANOVA followed by Dunnett's post hoc test.

(E) Menthol-induced analgesia. Left, experimental design. iDTR-expressing *Kcnip2*^{GlyT2} neurons was ablated through systemic DTX injection (0.1 mg/kg, i.p.). Right, paw withdrawal latencies in the Hargreaves test determined before and 24 h after subcutaneous zymosan A injection (0.06 mg in 20 μl saline) and 30 min after additional menthol injection (100 mg/kg, i.p.). n = 10 and 7, for control and *Kcnip2*^{GlyT2} ablated mice. Two-way ANOVA $F(1,16) = 1.2$, $p = 0.29$. Mean ± SEM.

responses. At present, we can only speculate about the underlying mechanisms, but circuits downstream of *Kcnip2*^{GlyT2} neurons may differentially affect heat and cold responses.

The specific increase in cold sensitivity after *Kcnip2*^{GlyT2} neuron ablation suggests either a preferential activation of dorsal horn *Kcnip2*^{GlyT2} neurons by input from cold-sensing fibers or a preferential inhibitory action of *Kcnip2* neurons on cold-specific dorsal horn output neurons (Hachisuka et al., 2016, 2020). As discussed above, our c-fos induction experiments suggest that cold activates dorsal horn *Kcnip2*^{GlyT2} neurons more efficiently than heat or mechanical stimuli. Conversely, the targeted recordings of lamina I projection neurons demonstrate that dorsal horn *Kcnip2*^{GlyT2} neurons inhibit lamina I projection neurons rather broadly. When taken together, both results suggest that the phenotype observed in *Kcnip2*^{GlyT2} neuron-ablated mice originates from preferential activation by cold input rather than from output specificity. Following this model, activation of *Kcnip2* neurons should have broader behavioral effects than the ablation of these neurons. In fact, activation of *Kcnip2*^{GlyT2} neurons also reduced heat responses and showed a trend toward reduced responses to painful (pin-prick) and nonpainful (brush) mechanical stimuli.

Inhibition of a relatively broad population of lamina I output neurons by *Kcnip2*^{GlyT2} neurons opens the possibility that *Kcnip2*^{GlyT2} neurons contribute to cooling-induced analgesia (Bini et al., 1984; Gammon and Starr, 1941; Kanui, 1985; Knowlton et al., 2013). Cooling-induced analgesia is not just a consequence of a rather nonspecific physical effect on peripheral nerve conduction but originates at least partly from central sites (for a review, see Foulkes and Wood, 2007). First, the inhibition of nocifensive reactions cannot only be induced by physical cooling but also by chemical activation of cold-sensitive sensory fibers with menthol or icilin (Liu et al., 2013; Proudfoot et al., 2006). Second, the suppression of nocifensive reactions in the formalin test caused by lowering the ambient temperature is reduced in mice with deficits in cold sensing (Dhaka et al., 2007). In our experiments, chemogenetic activation of spinal *Kcnip2*^{GlyT2} neurons suppressed nocifensive reactions in the formalin test. It also suppressed cooling-induced flinching behavior in mice with neuropathic cold allodynia, supporting that dorsal horn *Kcnip2*^{GlyT2} neurons contribute to cooling-induced analgesia.

Our electrophysiological experiments revealed that the vast majority of the *Kcnip2*^{GlyT2} neurons that receive input from icilin-sensitive fibers are located in lamina IIo and lamina I, that is, within the termination area of TRPM8-positive sensory fibers. This raises the question of whether the majority of *Kcnip2*^{GlyT2} neurons, which are located more deeply in laminae IIi and III, are also activated by cold. Interestingly, cold-induced c-fos expression in *Kcnip2*^{GlyT2} neurons was seen throughout the dorsal horn. Furthermore, previous morphological analyses have identified that much of the sensory input to *Kcnip2* neurons in the dorsal horn originates from rapidly adapting Aβ and Aδ, which are activated among other stimuli by local cooling (Abraira et al., 2017; Li et al., 2011). Both results support that dorsal horn *Kcnip2*^{GlyT2} neurons also receive input from cold-sensing fibers that do not express TRPM8. This scenario would also explain why ablation of *Kcnip2*^{GlyT2} neurons had no significant effect on menthol-induced analgesia. Although TRPM8 is considered the canonical sensor of environmental cold and cooling (Bautista et al., 2007; Colburn et al., 2007), strong evidence from several studies supports that it is not the only cold sensor. The number of cold-responsive neurons is reduced by only a little less than half in TRPM8-deficient mice (Colburn et al., 2007). The remaining cold-sensing neurons probably express other channels and receptors that contribute to cold sensing (Gong et al., 2019; Story et al., 2003; Viana et al., 2002; for a recent review, see MacDonald et al., 2020). Although unproven, some of the TRPM8-negative cold-sensing primary afferent fibers may terminate more deeply in the dorsal horn to activate *Kcnip2*^{GlyT2} neurons in laminae IIi and III. Such a scenario is also consistent with the strong cold sensitization observed after ablation of deep dorsal horn inhibitory neurons (Foster et al., 2015).

Cold allodynia is a common unpleasant symptom of neuropathic pain (Maier et al., 2010, for a review, see Scadding and Koltzenburg, 2013), whose circuit basis is only incompletely understood. While peripheral sensitizing processes certainly play a role, spinal mechanisms, which are well-established for mechanical allodynia (touch-evoked pain), may also contribute. Current concepts of mechanical allodynia are largely based on a loss of inhibition in the spinal dorsal horn, for example, through impaired chloride homeostasis (Coull et al., 2003) or loss of inhibitory synapses and interneurons (Moore et al., 2002). Such disinhibition unmasks normally silent polysynaptic pathways connecting non-nociceptive primary sensory fibers to spinal nociceptive output neurons (Hughes et al., 2012; Peirs and Seal, 2016; Peirs et al., 2015; Petitjean et al., 2015; Torsney and MacDermott, 2006). It is conceivable that a similar circuit exists for cold allodynia. Previous work has already suggested a form of gate control mechanism in which input from cooling sensitive Aδ fibers excites inhibitory dorsal horn neurons that in turn block input from nociceptive C fibers (Wasner et al., 2004). The Identification of *Kcnip2*^{GlyT2} neurons in the present report may therefore provide an entrance point for the direct investigation of central mechanisms of cold allodynia.

In summary, our results identify a population of spinal neurons that tune sensitivity to environmental cold. Some of the changes observed in mice after chemogenetic stimulation of these neurons suggest that they also contribute to cold-induced analgesia, while their dysfunction may lead to cold allodynia.

STAR★METHODS

Detailed methods are provided in the online version of this paper and include the following:

- KEY RESOURCES TABLE
- RESOURCE AVAILABILITY
  - Lead Contact
  - Materials Availability
  - Data and code availability
- EXPERIMENTAL MODEL AND SUBJECT DETAILS
  - Mice
  - Kcnip2^{roxCre} mice
  - Hoxb8::Dre transgenic mice
  - GlyT2::Dre BAC transgenic mice

CellPress
OPEN ACCESS

- ROSA26$^{roxSTOProx\text{-}tdTomato}$ reporter mice
- METHOD DETAILS
  - Southern blot
  - Immunohistochemistry and *in situ* hybridization
  - Intraspinal virus injections
  - Behavioral analyses
  - c-fos analysis of neuronal activation
  - Slice electrophysiology
- QUANTIFICATION AND STATISTICAL ANALYSIS

SUPPLEMENTAL INFORMATION

Supplemental information can be found online at https://doi.org/10.1016/j.neuron.2022.10.008.

ACKNOWLEDGMENTS

This work was supported by grants from the Swiss National Science Foundation (310030_197888), the University of Zurich (CRPP: "Pain—from phenotypes to mechanisms"), and a Wellcome Trust Collaborative Award in Science (F-41601-09-01). The authors thank Isabelle Kellenberger and Eva Roth for genotyping and mouse colony management.

AUTHOR CONTRIBUTIONS

G.W.A. generated the *Kcnip2^{roxCre}* targeting vector and performed morphological and behavioral experiments. R.P.G. performed the electrophysiological experiments and part of the behavioral tests. F.P., M.M.d.S., and K.W. contributed to the behavioral tests, and R.S. contributed to the morphological analyses. L.S. generated the *Hoxb8::Dre* and *GlyT2::Dre* targeting vectors. M.R.B. did the pronucleus injections of *GlyT2::Dre* and *Hoxb8::Dre* mice. P.P. provided an improved roxCre construct and performed embryonic stem cell (ES cell) injections. H.W. and H.U.Z. designed experiments together with G.W.A. and R.P.G. G.W.A., H.W., and H.U.Z. wrote the manuscript. All authors commented on and approved the manuscript.

DECLARATION OF INTERESTS

The authors declare no competing interests.

INCLUSION AND DIVERSITY

We support inclusive, diverse, and equitable conduct of research.

Received: October 8, 2021
Revised: August 16, 2022
Accepted: October 4, 2022
Published: November 1, 2022

REFERENCES

Abraira, V.E., Kuehn, E.D., Chirila, A.M., Springel, M.W., Toliver, A.A., Zimmerman, A.L., Orefice, L.L., Boyle, K.A., Bai, L., Song, B.J., et al. (2017). The cellular and synaptic architecture of the mechanosensory dorsal horn. Cell *168*, 295–310.e19.

Albisetti, G.W., Ghanem, A., Foster, E., Conzelmann, K.K., Zeilhofer, H.U., and Wildner, H. (2017). Identification of two classes of somatosensory neurons that display resistance to retrograde infection by rabies virus. J. Neurosci. *37*, 10358–10371.

Albisetti, G.W., Pagani, M., Platonova, E., Hösli, L., Johannssen, H.C., Fritschy, J.-M., Wildner, H., and Zeilhofer, H.U. (2019). Dorsal horn gastrin-releasing peptide expressing neurons transmit spinal itch but not pain signals. J. Neurosci. *39*, 102–117.

Bautista, D.M., Siemens, J., Glazer, J.M., Tsuruda, P.R., Basbaum, A.I., Stucky, C.L., Jordt, S.E., and Julius, D. (2007). The menthol receptor TRPM8 is the principal detector of environmental cold. Nature *448*, 204–208.

Behrendt, H.J., Germann, T., Gillen, C., Hatt, H., and Jostock, R. (2004). Characterization of the mouse cold-menthol receptor TRPM8 and vanilloid receptor type-1 VR1 using a fluorometric imaging plate reader (FLIPR) assay. Br. J. Pharmacol. *141*, 737–745.

Bini, G., Cruccu, G., Hagbarth, K.E., Schady, W., and Torebjörk, E. (1984). Analgesic effect of vibration and cooling on pain induced by intraneural electrical stimulation. Pain *18*, 239–248.

Braz, J.M., Rico, B., and Basbaum, A.I. (2002). Transneuronal tracing of diverse CNS circuits by Cre-mediated induction of wheat germ agglutinin in transgenic mice. Proc. Natl. Acad. Sci. USA *99*, 15148–15153.

Brenner, D.S., Golden, J.P., and Gereau, R.W.t. (2012). A novel behavioral assay for measuring cold sensation in mice. PLoS One *7*, e39765.

Buch, T., Heppner, F.L., Tertilt, C., Heinen, T.J., Kremer, M., Wunderlich, F.T., Jung, S., and Waisman, A. (2005). A Cre-inducible diphtheria toxin receptor mediates cell lineage ablation after toxin administration. Nat. Methods *2*, 419–426.

Colburn, R.W., Lubin, M.L., Stone, D.J., Jr., Wang, Y., Lawrence, D., D'Andrea, M.R., Brandt, M.R., Liu, Y., Flores, C.M., and Qin, N. (2007). Attenuated cold sensitivity in TRPM8 null mice. Neuron *54*, 379–386.

Coull, J.A., Boudreau, D., Bachand, K., Prescott, S.A., Nault, F., Sík, A., De Koninck, P., and De Koninck, Y. (2003). Trans-synaptic shift in anion gradient in spinal lamina I neurons as a mechanism of neuropathic pain. Nature *424*, 938–942.

Das Gupta, R.R., Scheurer, L., Pelczar, P., Wildner, H., and Zeilhofer, H.U. (2021). Neuron-specific spinal cord translatomes reveal a neuropeptide code for mouse dorsal horn excitatory neurons. Sci. Rep. *11*, 5232.

Dhaka, A., Earley, T.J., Watson, J., and Patapoutian, A. (2008). Visualizing cold spots: TRPM8-expressing sensory neurons and their projections. J. Neurosci. *28*, 566–575.

Dhaka, A., Murray, A.N., Mathur, J., Earley, T.J., Petrus, M.J., and Patapoutian, A. (2007). TRPM8 is required for cold sensation in mice. Neuron *54*, 371–378.

Dobrott, C.I., Sathyamurthy, A., and Levine, A.J. (2019). Decoding cell type diversity Within the spinal cord. Curr. Opin. Physiol. *8*, 1–6.

Ernst, E., and Fialka, V. (1994). Ice freezes pain? A review of the clinical effectiveness of analgesic cold therapy. J. Pain Symptom Manag. *9*, 56–59.

Fenno, L.E., Mattis, J., Ramakrishnan, C., Hyun, M., Lee, S.Y., He, M., Tucciarone, J., Selimbeyoglu, A., Berndt, A., Grosenick, L., et al. (2014). Targeting cells with single vectors using multiple-feature boolean logic. Nat. Methods *11*, 763–772.

Foster, E., Wildner, H., Tudeau, L., Haueter, S., Ralvenius, W.T., Jegen, M., Johannssen, H., Hösli, L., Haenraets, K., Ghanem, A., et al. (2015). Targeted ablation, silencing, and activation establish glycinergic dorsal horn neurons as key components of a spinal gate for pain and itch. Neuron *85*, 1289–1304.

Foulkes, T., and Wood, J.N. (2007). Mechanisms of cold pain. Channels *1*, 154–160.

Gammon, G.D., and Starr, I. (1941). Studies on the relief of pain by counterirritation. J. Clin. Invest. *20*, 13–20.

Gong, J., Liu, J., Ronan, E.A., He, F., Cai, W., Fatima, M., Zhang, W., Lee, H., Li, Z., Kim, G.H., et al. (2019). A cold-sensing receptor encoded by a glutamate receptor gene. Cell *178*, 1375–1386.e11.

Hachisuka, J., Baumbauer, K.M., Omori, Y., Snyder, L.M., Koerber, H.R., and Ross, S.E. (2016). Semi-intact ex vivo approach to investigate spinal somatosensory circuits. eLife *5*, e22866.

Hachisuka, J., Koerber, H.R., and Ross, S.E. (2020). Selective-cold output through a distinct subset of lamina I spinoparabrachial neurons. Pain *161*, 185–194.

Hachisuka, J., Omori, Y., Chiang, M.C., Gold, M.S., Koerber, H.R., and Ross, S.E. (2018). Wind-up in lamina I spinoparabrachial neurons: a role for reverberatory circuits. Pain *159*, 1484–1493.

Haenraets, K., Foster, E., Johannssen, H., Kandra, V., Frezel, N., Steffen, T., Jaramillo, V., Paterna, J.C., Zeilhofer, H.U., and Wildner, H. (2017). Spinal

nociceptive circuit analysis with recombinant adeno-associated viruses: the impact of serotypes and promoters. J. Neurochem. *142*, 721–733.

Häring, M., Zeisel, A., Hochgerner, H., Rinwa, P., Jakobsson, J.E.T., Lönnerberg, P., La Manno, G., Sharma, N., Borgius, L., Kiehn, O., et al. (2018). Neuronal atlas of the dorsal horn defines its architecture and links sensory input to transcriptional cell types. Nat. Neurosci. *21*, 869–880.

Hermann, M., Stillhard, P., Wildner, H., Seruggia, D., Kapp, V., Sánchez-Iranzo, H., Mercader, N., Montoliu, L., Zeilhofer, H.U., and Pelczar, P. (2014). Binary recombinase systems for high-resolution conditional mutagenesis. Nucleic Acids Res. *42*, 3894–3907.

Hole, K., and Tjølsen, A. (1993). The tail-flick and formalin tests in rodents: changes in skin temperature as a confounding factor. Pain *53*, 247–254.

Hughes, D.I., Sikander, S., Kinnon, C.M., Boyle, K.A., Watanabe, M., Callister, R.J., and Graham, B.A. (2012). Morphological, neurochemical and electrophysiological features of parvalbumin-expressing cells: a likely source of axo-axonic inputs in the mouse spinal dorsal horn. J. Physiol. *590*, 3927–3951.

Jensen, T.S., and Finnerup, N.B. (2014). Allodynia and hyperalgesia in neuropathic pain: clinical manifestations and mechanisms. Lancet Neurol. *13*, 924–935.

John, A., Wildner, H., and Britsch, S. (2005). The homeodomain transcription factor Gbx1 identifies a subpopulation of late-born GABAergic interneurons in the developing dorsal spinal cord. Dev. Dyn. *234*, 767–771.

Kanui, T.I. (1985). Thermal inhibition of nociceptor-driven spinal cord neurones in the rat. Pain *21*, 231–240.

Knowlton, W.M., Palkar, R., Lippoldt, E.K., McCoy, D.D., Baluch, F., Chen, J., and McKemy, D.D. (2013). A sensory-labeled line for cold: TRPM8-expressing sensory neurons define the cellular basis for cold, cold pain, and cooling-mediated analgesia. J. Neurosci. *33*, 2837–2848.

Li, L., Rutlin, M., Abraira, V.E., Cassidy, C., Kus, L., Gong, S., Jankowski, M.P., Luo, W., Heintz, N., Koerber, H.R., et al. (2011). The functional organization of cutaneous low-threshold mechanosensory neurons. Cell *147*, 1615–1627.

Liu, B., Fan, L., Balakrishna, S., Sui, A., Morris, J.B., and Jordt, S.E. (2013). TRPM8 is the principal mediator of menthol-induced analgesia of acute and inflammatory pain. Pain *154*, 2169–2177.

Lowitzsch, K., Hopf, H.C., and Galland, J. (1977). Changes of sensory conduction velocity and refractory periods with decreasing tissue temperature in man. J. Neurol. *216*, 181–188.

MacDonald, D.I., Wood, J.N., and Emery, E.C. (2020). Molecular mechanisms of cold pain. Neurobiol. Pain *7*, 100044.

Maier, C., Baron, R., Tölle, T.R., Binder, A., Birbaumer, N., Birklein, F., Gierthmühlen, J., Flor, H., Geber, C., Huge, V., et al. (2010). Quantitative sensory testing in the German Research Network on Neuropathic Pain (DFNS): somatosensory abnormalities in 1236 patients with different neuropathic pain syndromes. Pain *150*, 439–450.

McKemy, D.D., Neuhausser, W.M., and Julius, D. (2002). Identification of a cold receptor reveals a general role for TRP channels in thermosensation. Nature *416*, 52–58.

Miranda, C.O., Hegedüs, K., Wildner, H., Zeilhofer, H.U., and Antal, M. (2022). Morphological and neurochemical characterization of glycinergic neurons in laminae I–IV of the mouse spinal dorsal horn. J. Comp. Neurol. *530*, 607–626.

Moore, K.A., Kohno, T., Karchewski, L.A., Scholz, J., Baba, H., and Woolf, C.J. (2002). Partial peripheral nerve injury promotes a selective loss of GABAergic inhibition in the superficial dorsal horn of the spinal cord. J. Neurosci. *22*, 6724–6731.

Müller, T., Brohmann, H., Pierani, A., Heppenstall, P.A., Lewin, G.R., Jessell, T.M., and Birchmeier, C. (2002). The homeodomain factor lbx1 distinguishes two major programs of neuronal differentiation in the dorsal spinal cord. Neuron *34*, 551–562.

Pan, R., Tian, Y., Gao, R., Li, H., Zhao, X., Barrett, J.E., and Hu, H. (2012). Central mechanisms of menthol-induced analgesia. J. Pharmacol. Exp. Ther. *343*, 661–672.

Peier, A.M., Moqrich, A., Hergarden, A.C., Reeve, A.J., Andersson, D.A., Story, G.M., Earley, T.J., Dragoni, I., McIntyre, P., Bevan, S., et al. (2002). A TRP channel that senses cold stimuli and menthol. Cell *108*, 705–715.

Peirs, C., and Seal, R.P. (2016). Neural circuits for pain: recent advances and current views. Science *354*, 578–584.

Peirs, C., Williams, S.P., Zhao, X., Walsh, C.E., Gedeon, J.Y., Cagle, N.E., Goldring, A.C., Hioki, H., Liu, Z., Marell, P.S., et al. (2015). Dorsal horn circuits for persistent mechanical pain. Neuron *87*, 797–812.

Petitjean, H., Pawlowski, S.A., Fraine, S.L., Sharif, B., Hamad, D., Fatima, T., Berg, J., Brown, C.M., Jan, L.Y., Ribeiro-da-Silva, A., et al. (2015). Dorsal horn parvalbumin neurons are Gate-Keepers of touch-evoked pain after nerve injury. Cell Rep. *13*, 1246–1257.

Polgár, E., Sardella, T.C.P., Tiong, S.Y.X., Locke, S., Watanabe, M., and Todd, A.J. (2013). Functional differences between neurochemically defined populations of inhibitory interneurons in the rat spinal dorsal horn. Pain *154*, 2606–2615.

Poyatos, I., Ponce, J., Aragón, C., Giménez, C., and Zafra, F. (1997). The glycine transporter GLYT2 is a reliable marker for glycine-immunoreactive neurons. Brain Res. Mol. Brain Res. *49*, 63–70.

Proudfoot, C.J., Garry, E.M., Cottrell, D.F., Rosie, R., Anderson, H., Robertson, D.C., Fleetwood-Walker, S.M., and Mitchell, R. (2006). Analgesia mediated by the TRPM8 cold receptor in chronic neuropathic pain. Curr. Biol. *16*, 1591–1605.

Pruunsild, P., and Timmusk, T. (2012). Subcellular localization and transcription regulatory potency of KCNIP/Calsenilin/DREAM/KChIP proteins in cultured primary cortical neurons do not provide support for their role in CRE-dependent gene expression. J. Neurochem. *123*, 29–43.

Rodríguez, C.I., Buchholz, F., Galloway, J., Sequerra, R., Kasper, J., Ayala, R., Stewart, A.F., and Dymecki, S.M. (2000). High-efficiency deleter mice show that FLPe is an alternative to Cre-loxP. Nat. Genet. *25*, 139–140.

Rosland, J.H. (1991). The formalin test in mice: the influence of ambient temperature. Pain *45*, 211–216.

Sathyamurthy, A., Johnson, K.R., Matson, K.J.E., Dobrott, C.I., Li, L., Ryba, A.R., Bergman, T.B., Kelly, M.C., Kelley, M.W., and Levine, A.J. (2018). Massively parallel single nucleus transcriptional profiling defines spinal cord neurons and their activity during behavior. Cell Rep. *22*, 2216–2225.

Scadding, J.W., and Koltzenburg, M. (2013). Painful peripheral neuropathies. In Wall and Melzack's Textbook of Pain, A.P. McMahon, M. Koltzenburg, I. Tracey, and D. Turk, eds. (Saunders), pp. 926–951.

Schindelin, J., Arganda-Carreras, I., Frise, E., Kaynig, V., Longair, M., Pietzsch, T., Preibisch, S., Rueden, C., Saalfeld, S., Schmid, B., et al. (2012). Fiji: an open-source platform for biological-image analysis. Nat. Methods *9*, 676–682.

Shimizu, I., Iida, T., Guan, Y., Zhao, C., Raja, S.N., Jarvis, M.F., Cockayne, D.A., and Caterina, M.J. (2005). Enhanced thermal avoidance in mice lacking the ATP receptor P2X3. Pain *116*, 96–108.

Story, G.M., Peier, A.M., Reeve, A.J., Eid, S.R., Mosbacher, J., Hricik, T.R., Earley, T.J., Hergarden, A.C., Andersson, D.A., Hwang, S.W., et al. (2003). ANKTM1, a TRP-like channel expressed in nociceptive neurons, is activated by cold temperatures. Cell *112*, 819–829.

Szymczak-Workman, A.L., Vignali, K.M., and Vignali, D.A. (2012). Design and construction of 2A peptide-linked multicistronic vectors. Cold Spring Harb. Protoc. *2012*, 199–204.

Torsney, C., and MacDermott, A.B. (2006). Disinhibition opens the gate to pathological pain signaling in superficial neurokinin 1 receptor-expressing neurons in rat spinal cord. J. Neurosci. *26*, 1833–1843.

Viana, F., de la Peña, E., and Belmonte, C. (2002). Specificity of cold thermotransduction is determined by differential ionic channel expression. Nat. Neurosci. *5*, 254–260.

Wang, F., Li, E., De, L., Wu, Q., and Zhang, Y. (2021). OFF-transient alpha RGCs mediate looming triggered innate defensive response. Curr. Biol. *31*, 2263–2273.e3.

CellPress
OPEN ACCESS

Wasner, G., Schattschneider, J., Binder, A., and Baron, R. (2004). Topical menthol–a human model for cold pain by activation and sensitization of C nociceptors. Brain *127*, 1159–1171.

Wende, H., Lechner, S.G., Cheret, C., Bourane, S., Kolanczyk, M.E., Pattyn, A., Reuter, K., Munier, F.L., Carroll, P., Lewin, G.R., et al. (2012). The transcription factor c-Maf controls touch receptor development and function. Science *335*, 1373–1376.

Wildner, H., Das Gupta, R., Bröhl, D., Heppenstall, P.A., Zeilhofer, H.U., and Birchmeier, C. (2013). Genome-wide expression analysis of Ptf1a- and Ascl1-deficient mice reveals new markers for distinct dorsal horn interneuron populations contributing to nociceptive reflex plasticity. J. Neurosci. *33*, 7299–7307.

Witschi, R., Johansson, T., Morscher, G., Scheurer, L., Deschamps, J., and Zeilhofer, H.U. (2010). Hoxb8-Cre mice: A tool for brain-sparing conditional gene deletion. Genesis *48*, 596–602.

Wrigley, P.J., Jeong, H.J., and Vaughan, C.W. (2009). Primary afferents with TRPM8 and TRPA1 profiles target distinct subpopulations of rat superficial dorsal horn neurones. Br. J. Pharmacol. *157*, 371–380.

Zeilhofer, H.U., Studler, B., Arabadzisz, D., Schweizer, C., Ahmadi, S., Layh, B., Bösl, M.R., and Fritschy, J.M. (2005). Glycinergic neurons expressing enhanced green fluorescent protein in bacterial artificial chromosome transgenic mice. J. Comp. Neurol. *482*, 123–141.

Zeisel, A., Hochgerner, H., Lönnerberg, P., Johnsson, A., Memic, F., van der Zwan, J., Häring, M., Braun, E., Borm, L.E., La Manno, G., et al. (2018). Molecular architecture of the mouse nervous system. Cell *174*, 999–1014.e22.

STAR★METHODS

KEY RESOURCES TABLE

REAGENT	SOURCE	IDENTIFIER
**Antibodies**		
goat anti-Pax2 (1:200)	R and D Systems	Cat#AF3364; RRID:AB_10889828
goat anti-CGRP (1:500)	Abcam	Cat#ab36001; RRID:AB_725807
goat anti-tdTomato (1:1000)	SICGEN	Cat# AB8181-200; RRID:AB_2722750
goat anti-TrkA (1:400)	R & D Systems	Cat#AF1056; RRID:AB_2283049
goat anti-WGA	Vector Laboratories	Cat#AS-2024; RRID:AB_2315608
guinea pig anti-Lmx1b (1:10.000)	Müller et al., 2002	N/A
mouse anti-NeuN (1:500)	Millipore	Cat#MAB377; RRID:AB_2298772
rabbit anti-cfos (H125) (1:500)	Santa Cruz Biotechnology	Cat#SC7202; RRID:AB_2106765
rabbit anti-Cmaf (1:10.000)	Wende et al., 2012	N/A
rabbit anti-Gbx1 (1:5000)	John et al., 2005	N/A
rabbit anti-GFP (1:1000)	Molecular Probes	Cat#A6455; RRID:AB_221570
Rabbit anti-NeuN (1:3000)	Abcam	Cat#ab104225; RRID:AB_10711153
rabbit anti-NF200 (1:1000)	Sigma	Cat#N4142; RRID:AB_477272
rabbit anti-P2X3 (1:1000)	Abcam	Cat#ab10269; RRID:AB_297006
rabbit anti-PKCγ (1:1000)	Santa Cruz Biotechnology	Cat#sc-211; RRID:AB_632234
rabbit anti-PV (1:2000)	Swant	Cat#PV25; RRID:AB_10000344
rabbit anti-WGA (1:2000)	Sigma-Aldrich	Cat#T4144; RRID:AB_261669
sheep anti-PlexinC1 (1:400)	R & D Systems	Cat#AF5375; RRID:AB_2284038
sheep anti-TH (1:1000)	Millipore	Cat# AB1542; RRID:AB_90755
Alexa Fluor 488-donkey anti-goat (1:800)	Jackson ImmunoResearch	Cat#795-546-147; RRID:AB_2340430
Alexa Fluor 488-donkey anti-rabbit (1:800)	Jackson ImmunoResearch	Cat#711-546-152; RRID:AB_2340619
Alexa Fluor 647-donkey anti-goat (1:800)	Jackson ImmunoResearch	Cat#705-605-147; RRID:AB_2340437
Alexa Fluor 647-donkey anti-guinea pig (1:800)	Jackson ImmunoResearch	Cat#706-496-148; RRID:AB_2340477
Alexa Fluor 647-donkey anti-rabbit (1:800)	Jackson ImmunoResearch	Cat#711-607-003; RRID:AB_2340626
DyLight™ 647-donkey anti-sheep (1:800)	Jackson ImmunoResearch	Cat#713-606-147; RRID:AB_2340752
**Fluorescent conjugates**		
Alexa Fluor 647-Isolectin GS-IB4 (1:500)	Thermo Fisher Scientific	Cat#I32450; RRID:SCR_014365
**Bacterial and virus strains**		
AAV1.EF1a.flex.DTA (3.3 × 10^{12} IU/ml)	Foster et al., 2015	N/A
AAV1.hSyn.flex.hM3Dq-mCherry (6.5 × 10^{12} IU/ml)	Foster et al., 2015	N/A
AAV2.Ef1a.flex.WGA (5.3 × 10^{12} IU/ml)	UNC Vector Core	N/A
AAV1.Ef1a.flex.eGFP (4.7 × 10^{12} IU/ml)	VVF Zurich	v217
**Chemicals, peptides, and recombinant proteins**		
clozapine-N-oxide (CNO)	Enzo Life Sciences	BBL-NS105-0025
DTX	Millipore	Cat. No. 322326
icilin	Sigma	i9532-10mg
(-)-menthol	Merck	Cat. No. 63660-100G
**Critical commercial assays**		
Mm-Kcnip2-C3	Advanced Cell Diagnostics	Cat No. 536361-C3
Mm-Slc32a1-C2	Advanced Cell Diagnostics	Cat No. 319191-C2
tdTomato	Advanced Cell Diagnostics	Cat No. 317041

(*Continued on next page*)

Continued		
REAGENT	SOURCE	IDENTIFIER
RNAscope Fluorescent Multiplex Reagent Kit	Advanced Cell Diagnostics	Cat No. 320850
Deposited data		
Raw data	This paper	https://doi.org/10.5281/zenodo.7143887
Experimental models: Organisms/strains		
Mouse: *GlyT2::Dre*	This paper	N/A
Mouse: *Hoxb8::Dre*	This paper	N/A
Mouse: $Kcnip2^{roxCre}$	This paper	N/A
Mouse: $ROSA26^{lsl\text{-}iDTR}$	The Jackson Laboratory	RRID:IMSR_JAX:007900
Mouse: $ROSA26^{lsl\text{-}tdTom}$	The Jackson Laboratory	RRID:IMSR_JAX:007914
Mouse: $ROSA26^{rsr\text{-}tdTom}$	This paper	Derived from RRID:IMSR_JAX:021876
Software and algorithms		
GraphPad Prism version 5	GraphPad Software	https://www.graphpad.com/scientific-software/prism/
Fiji ImagJ	Schindelin et al., 2012	https://imagej.net/software/fiji/
CineLAB	Plexon Inc.	https://plexon.com/cinelab-video-tracking-system/

RESOURCE AVAILABILITY

Lead Contact

Further information and requests for resources and reagents should be directed to and will be fulfilled upon reasonable request by lead contact, Hanns Ulrich Zeilhofer, (zeilhofer@pharma.uzh.ch).

Materials Availability

The transgenic mouse lines *GlyT2::Dre*, *Hoxb8::Dre*, and $Kcnip2^{roxCre}$ are available upon reasonable request after signing a material transfer agreement with the University of Zurich.

Data and code availability

- Raw data have been deposited at ZENODO.org and are publicly available as of the date of publication. DOIs are listed in the key resources table.
- This paper does not report original code
- Any additional information required to reanalyze the data reported in this paper is available from the lead contact upon request.

EXPERIMENTAL MODEL AND SUBJECT DETAILS

Mice

Permissions for animal experiments have been obtained from the Canton of Zurich. All procedures followed the ethical guidelines of the University of Zurich. Apart for the thermal gradient test, in which only male mice were included, all other experiments were performed on both male and female mice. All behavioral tests and morphological analyses were performed on adult 6 - 12-week old mice. Mice used in electrophysiological experiments were aged 4 - 8 weeks. Because of low statistical power no sex specific analyses were made. None of mice reported in this paper have been used in previous studies and were drug/test naïve.

$Kcnip2^{roxCre}$ mice

Wild-type and targeted *Kcnip2* loci are illustrated in Figure S1A. The construct used to generate $Kcnip2^{roxCre}$ knock-in mice was obtained through repeated enzymatic digestions and fragment ligations performed on a pBluescript cloning vector. The generated construct incorporated the sequence of an optimized roxed-Cre recombinase sequence preceded by the sequence coding for a short ‘‘self-cleaving’’ peptide *P2A*, and followed by an FRT-flanked neomycin resistance gene (*Neo*) (Hermann et al., 2014; Szymczak-Workman et al., 2012). Homologous recombination in SW102 cells was used to insert the generated construct into pDTA plasmid previously equipped with 17.2 kb of *Kcnip2* gene sequence from BAC clone CH29-98H02. Homologous recombination was guided towards the third coding exon of *Kcnip2* gene (exon common to all known *Kcnip2* splice variants; Pruunsild and Timmusk, 2012). After confirming successful recombination using restriction digest reactions and DNA Sanger sequencing, the construct was

linearized and purified. The construct was electroporated into mouse embryonic stem cells (ES cells) at the Institute of Laboratory Animal Science of the University of Zurich. Duplicate samples of ES cells were screened for appropriate gene recombination by Southern blot. Positive clones were injected into the blastocyst of *albino*-C57Bl/6 mice to generate chimeric mice. Chimeric mice were crossed with C57Bl/6 wild-type mice to generate *Kcnip2*^{roxCre} heterozygous mice and with *ACTB::FLPe* mice to excise the Neo sequence (Rodríguez et al., 2000). Polymerase chain reaction (PCR) using the primers GGT TCA GAC CTT CTG GCG TG and TGC ACA CAG ACA GGA GCA TCT TC was used to confirm the presence of roxed-Cre in the mouse genome. The presence of the *Kcnip2* wild-type allele was tested using GGG CCT GAC CTG AGG CAA AG and AGC AGA CCT CTT CCC CGT GT primers.

Hoxb8::Dre transgenic mice

Hoxb8::Dre mice were generated as described previously for *Hoxb8::Cre* mice (Witschi et al., 2010; for details see Figure S2). Dre-dependent recombination was characterized using a Dre dependent reporter mouse line ROSA26$^{roxSTOProx\text{-}tdTomato}$ (*ROSA26*$^{rsr\text{-}tdTom}$). The presence of the *Hoxb8::Dre* transgene was confirmed by PCR using the primers GCC TCA AAA TTC AAT AAA ACG CCA C and CAG GTC TCC CAC TCT GAT CCT AG.

GlyT2::Dre BAC transgenic mice

GlyT2::Dre mice were generated using the same strategy as previously applied to generate *GlyT2::eGFP* (Zeilhofer et al., 2005), *GlyT2::Cre* (Foster et al., 2015), and *GlyT2::Cre*^{ERT2} (Miranda et al., 2022) mice (for details see Figure S4). Proper expression of a Dre-dependent reporter gene was verified using a Dre-dependent reporter mouse line (*ROSA26*$^{rsr\text{-}tdTom}$) (Figure S4). The *GlyT2::Dre* transgene was detected by PCR using the primers ATG TGT GCA TCT GTG TAT GCA GAC C and CAG GTC TCC CAC TCT GAT CCT AG.

ROSA26$^{roxSTOProx\text{-}tdTomato}$ reporter mice

The Dre-dependent reporter mouse line *ROSA26*$^{rsr\text{-}tdTom}$ was generated by crossing Ai66D (Gt(ROSA)26Sor$^{tm66.1(CAG\text{-}tdTomato)Hze}$) mice to *EIIa::Cre* (Tg(EIIa-cre)C5379Lmgd/J) (PMCID: PMC39152) mice. The offspring were back crossed to C57Bl/6J mice to remove the *EIIa::Cre* transgene.

METHOD DETAILS

Southern blot

One to 5 μg of ES cells genomic DNA (gDNA) were digested using EcoR I or Bgl II restriction enzymes and then separated overnight at 25 V in 0.8% agarose gel. After equilibration of the gel for 20 min in 5 × SSC with 0.25 M NaOH and two times 10 min in 5 × SSC buffer with 10 mM NaOH, the DNA was transferred onto a Nylon membrane (0.45 μm pore size) using a vacuum blotter. The nylon membrane was briefly rinsed in 5 × SSC before UV crosslinking. Hybridization was performed using biotinylated probes generated using TCC ATT TGA TAG CAT CCT AAG CTG TAA and ATA TGT GTG TGT GTG TGA ATC TGT GTG primers for 5' external probe (*5'ext*) and GGA TCG GCC ATT GAA CAA GAT GG and GAT CCC CTC AGA AGA ACT CGT C primers for the *Neo* probe. After 60 min pre-incubation at 55°C in hybridization wash solution (20% ethylene carbonate, 0.25 M TEA-Cl, 0.1 × SSC, 0.1% SDS, pH 7.5) supplemented with 0.2 mg / ml fish DNA, 50 ng / ml biotine-labelled probes were added to the solution and incubated overnight at 55°C in a rotating hybridization oven. The nylon membrane was finally washed two times for 10 min each in hybridization wash solution and once for 5 min in SSPE. Detection was performed as described in KPL AP Chemiluminescent Blotting Kit (catalog number 54-30-01). Correct recombination was confirmed by the presence of 14 kb and 11 kb EcoR I-digested fragments using *5'ext* probe and by the presence of a 11 kb Bgl II-digested fragment using *Neo* probe (Figure S1B).

Immunohistochemistry and *in situ* hybridization

Antibody staining of brain and spinal cord tissue for neurochemical characterization of *Kcnip2* neurons was performed on free floating sections. DRG and spinal cord tissues prepared from mice used in behavioral and tracing experiments were immunostained on glass slides. Antibodies used are listed in the key resources table. For details on the protocol used for immunohistochemical reactions (Albisetti et al., 2019). For *in situ* hybridization of mRNA, tissue was prepared as described in Das Gupta et al. (2021) and Albisetti et al. (2019) and hybridized using probes designed for RNAscope® Fluorescent Multiplex in situ hybridization following RNAscope® Assay guidelines (Advanced Cell Diagnostics, Newark, CA, USA). Probes used are listed in the key resource table.

Intraspinal virus injections

Intraspinal virus injections were made in isoflurane-anesthetized mice using a motorized stereotaxic frame (David Kopf Instruments and Neurostar). Three separate unilateral injections of 300 nl each were carried out at a depth of 300 μm using glass micropipettes connected to a PHD ultra nanomite syringe pump (Harvard Apparatus) as described previously (Albisetti et al., 2017; Haenraets et al., 2017).

Behavioral analyses

Mice were kept at 12:12 h light/dark cycle and received food and water *ad libitum*. All behavioral tests were performed during the light phase (10.00 AM (ZT = 03) and 04.00 PM (ZT = 09) with the observer blinded to the genotype (or treatment) of the mice. For

nociception assays, mice were placed into plexiglas boxes and allowed to adapt to the new environment for 30 min. In chemogenetic experiments, behavioral tests were performed 1 - 2 weeks after AAV1.hsyn.flex.hM3dq.mCherry injection. Measurements were taken starting 1 hr after i.p. injection of CNO (2 mg/kg). Baselines were recorded immediately before CNO administration.

Somatosensory tests

Mechanical withdrawal thresholds were assessed using an electronic von Frey anesthesiometer (IITC) and the maximal force applied on the mouse paw was restricted to 6 g. Withdrawal latencies to noxious heat were assessed using Hargreaves test apparatus (IITC) with a temperature controlled glass platform set to 30°C. Withdrawal latencies to noxious cold were assessed cooling the 5 mm thick borosilicate glass platform directly under the mouse hind paw using a cold probe (powdered dry ice compressed into a 1 cm large syringe) (Brenner et al., 2012). For Hargreaves and cold tests, cut-off times were set to 32 s and 24 s, respectively. For von Frey, Hargreaves and cold tests, 8 measurements were taken per hind paw in 5 - 10 min intervals and average hind paw withdrawal thresholds were calculated for each mouse. For pin-prick test and brush tests, measurements were taken by stimulating the plantar surface of the mouse hind paw with a blunted G26 needle and a soft paint brush, respectively. Eight measurements were taken at an interval of 2 min and responses were scored as “0” for no reaction or “1” if the mouse responded.

Rotarod test

Motor coordination was tested using a rotarod instrument that linearly accelerated from 0 to 40 rpm over 5 min, and the latency to fall was measured for each mouse. Two training sessions were performed before the test experiment. Six measurements were taken per mouse.

Thermal gradient test

Tests were performed 7 to 8 days following DTX (0.1 mg/kg, i.p.) administration in *Kcnip2*^{GlyT2}-iDTR mice. Mice were placed on a custom built 84.5 cm long and 8.5 cm wide metal plate and temperatures of 13°C and 39°C were maintained at the extremities. The thermal gradient plate was virtually divided into 16 zones of equal size. For additional analyses, 5 to 6 zones were combined to represent cold (15 - 22°C), cool (22 - 28°C), and warm (28 - 39°C) areas. The activities of individual mice were tracked for 60 min using CineLAB^{TM} video tracking system (Plexon Inc). The median preferred temperature was calculated as the temperature below and above which mice spent 50% of their time on the thermal gradient plate.

Formalin test

Responses to intraplantar injection of formalin (5% w/v dissolved in 0.9% saline) were assessed *Kcnip2*^{roxCre};*GlyT2::dre* double transgenic mice, or control mice lacking one or both of these transgenes received intraspinal injections of AAV1.hsyn.flex.hM3dq.mCherry into the left lumbar spinal cord. At least 10 days after intraspinal injection, animals received an i.p. injection of CNO (2 mg/kg) 1 hour before testing. The left plantar surface of hind paws were injected with 20 µl freshly depolymerised paraformaldehyde (5% w/v) using a custom-made hindlimb restrainer. Mice were placed on a temperature-controlled platform heated to 30°C to ensure that cooling-sensitive afferents of the hindlimbs were inactive during testing. Mice were placed in transparent Plexiglas boxes and injected with CNO. Formalin was injected 60 min later. Mice were filmed from two different angles for 45 min following formalin injection. Time spent licking the ipsilateral paw was measured and grouped into 5 min epochs, with the experimenter being blinded to the animal genotype during the scoring.

Menthol-induced analgesia

Tests were performed 7 to 8 days following DTX (0.1 mg/kg) i.p. administration in *Kcnip2*^{GlyT2}-iDTR mice. Inflammatory hyperalgesia was induced through subcutaneous injection of zymosan A (0.06 mg in 20 µl) into the plantar surface of the left hindpaw. Twenty four hours later, mice were treated with (-)menthol (100 mg/kg, i.p.). Noxious heat sensitivity was measured in the Hargreaves test at baseline (after neuron ablation, before zymosan), 24 hours post zymosan, and 30 min post menthol injection.

Chronic constriction injury

Three loose (5-0, not absorbable) silk (Ethicon) ligatures were applied to the left sciatic nerve proximal to the trifurcation in mice anesthetized with isoflurane 1 - 3 %. Skin was closed using 5-0 Dermalon suture (Covidien).

Cold allodynia measurements

Cold allodynia was assessed using the acetone test. Mice were place on a mesh grid in plexiglass boxes and were allowed to adapt to the setup for at least 30 min. the plantar surface of the hindlimbs were sprayed with room temperature acetone using a 1 ml syringe. Flinches and shaking bouts were counted for a 1 min period after stimulation. Four Stimuli were given per time point, with a recovery period of 15 min between tests.

Skin temperature measurements

An infra-red thermometer was used to measure skin temperature at the lower abdomen.

c-fos analysis of neuronal activation

To test stimulus dependent activation of spinal neurons mice were briefly anesthetized using 1 - 2.5% isoflurane. The cold stimulus was applied by dropping -20°C cold acetone onto the plantar surface of the left hind paw at a rate of about one drop per second for 3 min (Häring et al., 2018). The heat stimulus was applied by immersing the left hind paw in water at 52°C for 20 s. The noxious mechanical stimulus was applied by repeatedly pinching folds of skin (6 each on the dorsal and ventral surface of the hind paw, applied with forceps for 5 s at each point over the course of 1 min) (Polgár et al., 2013). Control mice were anesthetized for 3 min but did not receive any hind paw stimulation.

Slice electrophysiology

Transverse spinal cord slices were taken from the lumbar enlargement of the spinal cord similarly to previous studies. In brief, mice were decapitated and the vertebral column was quickly dissected and placed into ice-cold oxygenated dissecting solution (containing in mM: 65 NaCl, 105 sucrose, 2.5 KCl, 1.25 NaH_2PO_4, 25 $NaHCO_3$, 25 glucose, 0.5 $CaCl_2$, 7 $MgCl_2$). The vertebral column was pinned to a sylgard coated dish with the ventral side facing up, and the vertebrae were removed. The spinal cord was carefully removed from the vertebral column and the lumbar enlargement was isolated from the rest of the cord. The lumbar tissue was glued to an agar block from the ventral side using superglue, which was then mounted in a slicing chamber that was installed on a vibrating blade microtome (D.S.K microslicer DTK1000). Transverse slices were then cut at 300 - 350 μm thickness in ice cold oxygenated dissecting solution, and were transferred to an incubation chamber filled with warm (34°C) oxygenated aCSF (containing in mM: 120 NaCl, 26 $NaHCO_3$, 1.25 NaH_2PO_4, 2.5 KCl, 5 HEPES, 14.6 glucose, 2 $CaCl_2$, 1 $MgCl_2$; pH 7.35 - 7.40, osmolarity 305 - 315 mOsm). Slices were allowed to adapt for 30 min prior to recording. Signals were acquired with a HEKA EPC10 amplifier and were recorded using patchmaster software at a sampling frequency of 20 kHz (HEKA Elektronik).

For spontaneous EPSC recordings, targeted whole-cell recordings were made from tdTom expressing neurons in slices taken from *Kcnip2*^{roxCre};*GlyT2::dre*;ROSA26^{tdTom} triple transgenic animals. A Cs-based internal solution was used for voltage clamp recordings (containing in mM: 120 CsCl, 10 HEPES, 0.05 EGTA, 2 $MgCl_2$, 2 Mg-ATP, 0.1 Na-GTP, 5 QX-314) and *Kcnip2*^{GlyT2} neurons were recorded from a holding potential of -70 mV. Slices were perfused at a rate of 2 - 3 ml/min with aCSF prewarmed to 32°C using an inline heater (Warner Instruments). This recording temperature was chosen to ensure low activity of cooling-sensitive afferent terminals in the dorsal horn (Wrigley et al., 2009). Tissues were perfused with bicuculline (20 μM) and strychnine (0.5 μM) for at least 3 min to block inhibitory synaptic events, and a 2 min recording was taken for baseline spontaneous EPSC activity. Slices were then perfused with icilin (20 μM) for at least 3 min, and a second recording of 2 min was taken. Spontaneous EPSC events were counted manually using MiniAnalysis software (Synaptosoft).

Light-evoked activity of *Kcnip2*^{GlyT2} neurons was assessed in *Kcnip2*^{roxCre};*GlyT2::dre*;*ROSA26*$^{ChR2-YFP}$ triple transgenic animals. Blue-light activation of *Kcnip2* neurons was confirmed by taking whole-cell recordings from ChR2-YFP-expressing cells using a K-gluconate internal solution (containing in mM, 130 K-gluconate, 5 NaCl, 1 EGTA, 10 HEPES, 5 Mg-ATP, 0.5 Na-GTP). Cells were recorded in current clamp mode, and 4 ms or 1 s blue light pulses were delivered using a monochromator (TILL photonics).

For targeted recordings from spinoparabrachial neurons, *Kcnip2*^{roxCre};*GlyT2::dre*;*ROSA26*$^{ChR2-YFP}$ mice received bilateral injections of AAV2retro.tdTom into the parabrachial areas (-5.2, +/-1.2, 4) relative to bregma (500 nl per injection) at least 10 days prior to recording experiments. Whole-cell recordings were taken from tdTom expressing neurons in lamina I using CsCl internal solution, with recorded cells being maintained at a holding potential of -70 mV. Blue light pulses (473 nm, 4 ms) were delivered at 0.1 Hz through the objective lens from the monochromator, and post-synaptic responses were measured in the recorded cells. Cells were considered to receive monosynaptic inhibitory synapses from *Kcnip2*^{GlyT2} neurons if synaptic events were reliably evoked without failure for 10 consecutive pulses, which were subsequently blocked by bath application of bicuculline (20 μM) and strychnine (0.5 μM).

Access resistance was monitored throughout recordings and data were excluded if this increased by >30% during the recording. Data were analysed in Igor Pro software using the PPT plugin (WaveMetrics, Inc.).

QUANTIFICATION AND STATISTICAL ANALYSIS

All statistical comparisons were made using unpaired t tests (GraphPad Prism version 5 for Windows, GraphPad Software). To analyze the horizontal curve-shift in thermal preference, medians of the areas under the curve (AUC) were calculated for each individual mouse and groups were compared using unpaired t test (two statistically significant outliers were excluded from the analysis). For cooling-induced analgesia experiments, data were analyzed using a One-Way ANOVA with Dunnett's post hoc test, with measurements post-nerve injury being considered the comparison group. To determine an altered distribution in the inter-event intervals between spontaneous EPSC, a Kolmogorov-Smirnov test was used. All data are given as mean ± SEM. $P \leq 0.05$ were considered statistically significant. n numbers and their specification are reported in the figure legends.

Neuron, Volume *111*

Supplemental information

Inhibitory *Kcnip2* neurons of the spinal dorsal horn control behavioral sensitivity to environmental cold

Gioele W. Albisetti, Robert P. Ganley, Francesca Pietrafesa, Karolina Werynska, Marília Magalhaes de Sousa, Rebecca Sipione, Louis Scheurer, Michael R. Bösl, Pawel Pelczar, Hendrik Wildner, and Hanns Ulrich Zeilhofer

Inhibitory *Kcnip2* neurons of the spinal dorsal horn control behavioral sensitivity to environmental cold

Gioele W. Albisetti, Robert Ganley, Francesca Pietrafesa, Karolina Werynska, Marília Magalhaes de Sousa, Rebecca Sipione, Louis Scheurer, Michael R. Bösl, Pawel Pelczar, Hendrik Wildner, Hanns Ulrich Zeilhofer

Supplemental figures

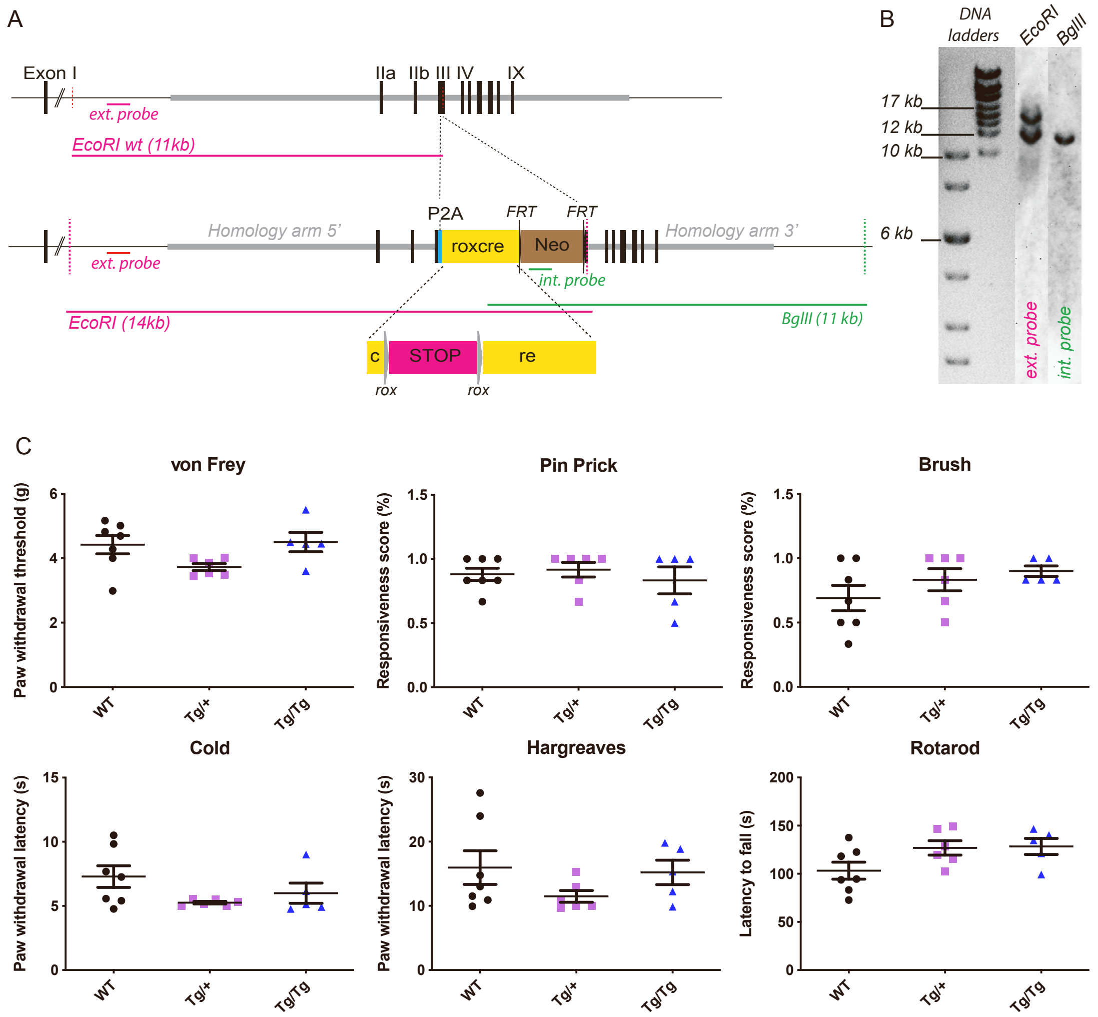

Fig. S1: Generation of *Kcnip2*^{roxCre} knock-in mice, Related to Figure 1 and STAR Methods. A, Scheme showing the targeting strategy for the generation of *Kcnip2*^{roxCre} mouse line. Homologous recombination was used to insert the cloned construct containing *P2A*, *roxCre* and *Neo* sequences into exon III of *Kcnip2* gene. The *Neo* sequence has been removed in a second step through cross breeding with *ACTB:FLPe* mice (not shown). Probes, restriction sites, length of the fragments generated, and the probes used for southern blot analysis are indicated in the figure. **B**, Southern blot result obtained from a clone with correct integration of the cloned construct. gDNA digested with EcoR I restriction enzyme and hybridized using *5'ext* probe results in a labelling of two bands of 14 kb and 11 kb in length, whereas gDNA digested with Bgl II restriction enzyme and hybridized using *Neo* probe labels a single DNA fragment of 11 kb. **C**, Sensory responses in wild-type mice (n = 7) and heterozygous (n = 6) and homozygous *Kcnip2*^{roxCre} mice (n = 5). One-way ANOVA: von Frey $F(2,15) = 2.902$, $P = 0.0860$; pin prick $F(2,15) = 0.3429$, $P = 0.7151$; brush $F(2,15) = 1.576$, $P = 0.2393$, cold $F(2,15) = 2.427$, $P = 0.1221$, Hargreaves $F(2,15) = 1.379$, $P = 0.2821$; rotarod $F(2,15) = 2.993$, $P = 0.0806$. Mean ± SEM.

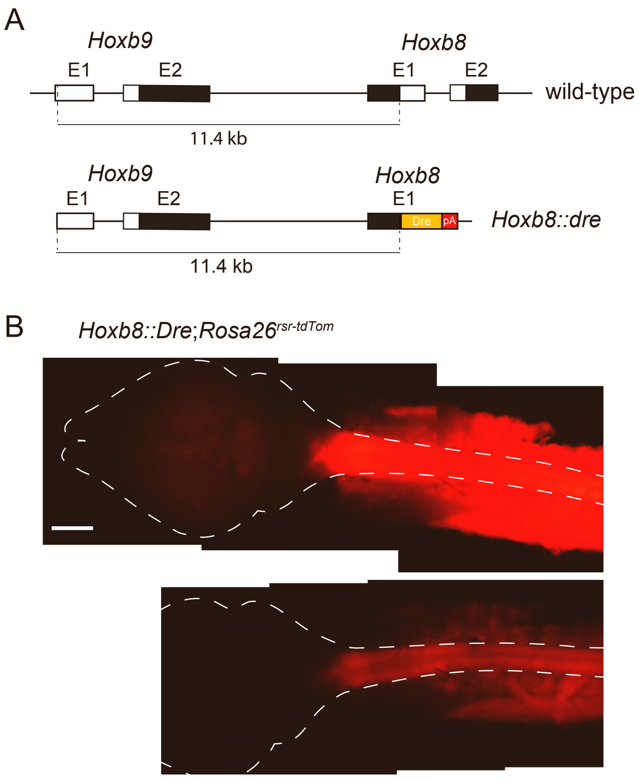

Fig. S2: Generation and gross characterization of Hoxb8::Dre BAC transgenic mice, Related to Figure 1 and STAR Methods. A, Genomic structure of the murine wild-type *HoxB8* gene locus. The 11.4 kb genomic fragment that has been used to drive expression of the Dre transgene is indicated. The *Hoxb8::Dre* transgene contains the 11.4 kb genomic fragment including the annotated *Hoxb8* promoter and a Dre expression cassette in frame with the ATG of the *Hoxb8* gene. (B,C) Expression analysis of the recombination pattern that occurred in *Hoxb8::Dre;Rosa26$^{rsr\text{-}tdTom}$* double transgenic mice. **B**, Widespread tdTom expression was observed in the CNS (outlined by dotted white line) and other tissues in the trunk of double transgenic animals. No tdTom expression was observed at supraspinal levels. Top and bottom micrographs show the same tissue but were obtained with different exposure times. Scale bars, 1 mm.

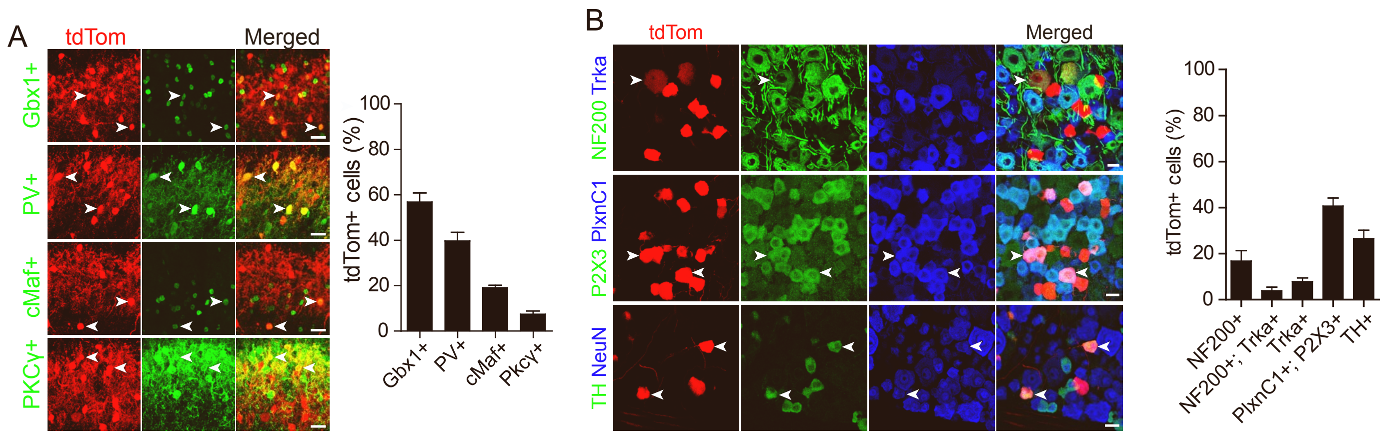

Fig. S3: Neurochemical characterization of *Kcnip2*^{Hoxb8} neurons, Related to Figure 1. Lumbar dorsal horn (A) and lumbar DRGs (B). Percent tdTom positive cells expressing the respective marker. n = 9 sections from a total of 3 mice (A) and n = 6 sections from 2 mice (B). Scale bars, 20 µm. Mean ± SEM.

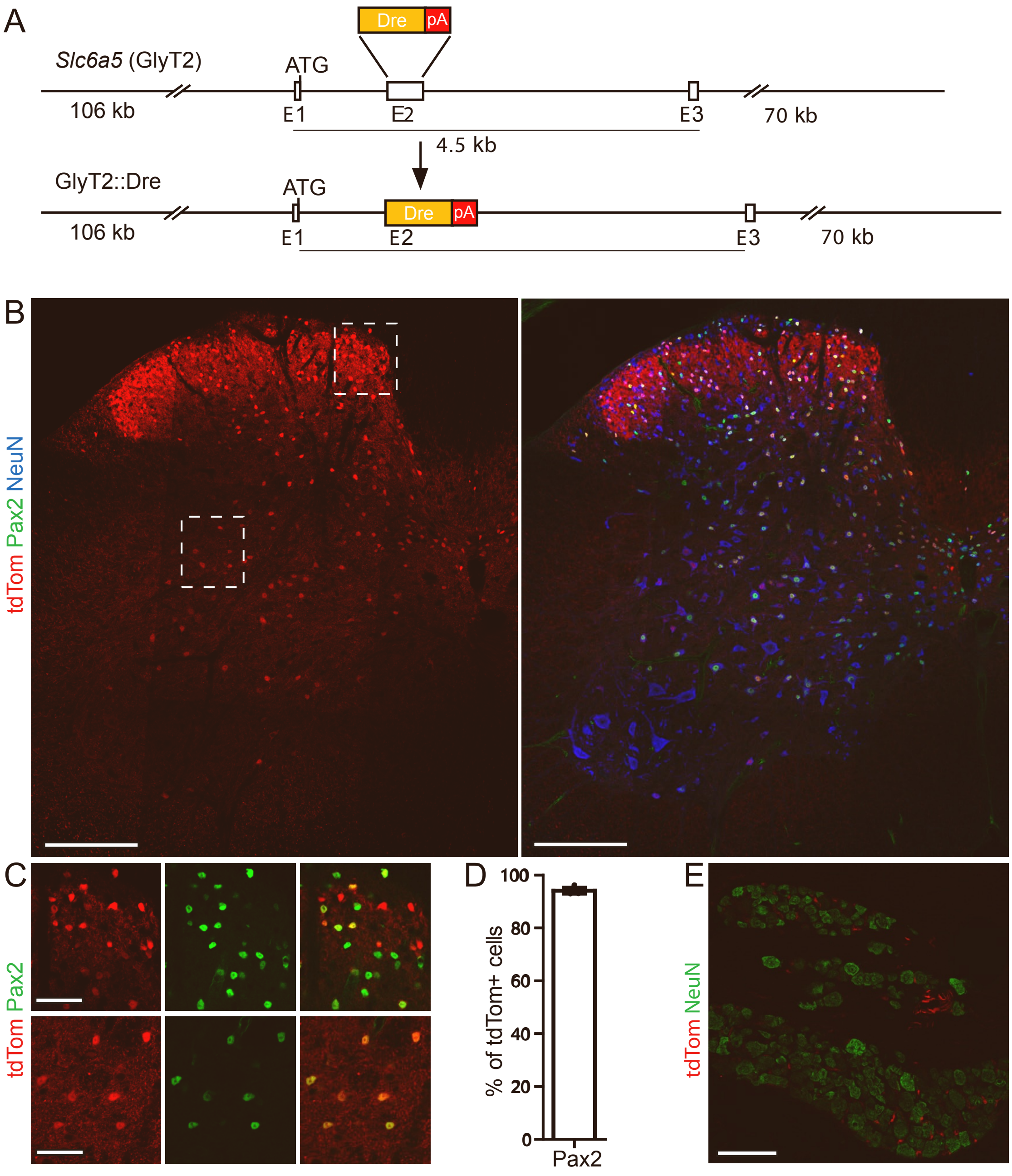

Fig. S4: Generation and characterization of BAC transgenic *GlyT2::Dre* mice, Related to Figure 2 and STAR Methods. A, Schematic representation of the generation of the *GlyT2::Dre* allele. The *GlyT2::Dre* coding sequence was placed into the second exon of the GlyT2 gene that starts with the second coding triplet. **B**, Immunohistochemistry showing the overlap of tdTom, Pax2 and NeuN in lumbar spinal cord sections of *GlyT2::Dre* mice crossed with Rosa26$^{rsr\text{-}tdTom}$ reporter mice. **C**, Insets from (B) showing the overlap between tdTom and Pax2. **D**, Quantification of (C) (913 tdTom+ neurons from n = 3 mice) **E**, No expression of tdTom in the DRG of *GlyT2::Dre* mice crossed with Rosa26$^{rsr\text{-}tdTom}$ reporter mice. Scale bars: B, 200 µm; C, 20 µm; E,100 µm. Mean ± SEM.

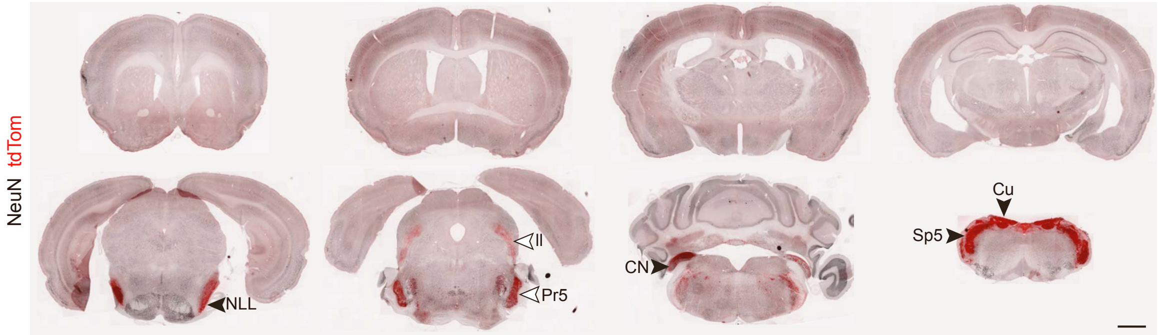

Fig. S5: Supraspinal *Kcnip2* expression, Related to Figure 1. Outside the spinal cord, *Kcnip2*^{GlyT2}-tdTom neurons were observed only in the hindbrain, including the brainstem, in particular in the spinal trigeminal nuclei (Sp5) and in the cuneate nucleus (Cu), which receive somatosensory information from the face and upper body, respectively. In addition, neurons were detected along the auditory pathways in the cochlear nucleus (CN) and in the nuclei of the lateral lemniscus (NLL), which are structures that receive and process acoustic information coming from the inner ear. tdTom positive fibers, but not cell bodies, were detected in principal sensory nucleus (Pr5) and in the lateral lemniscus (ll). Forebrain and cortical regions were devoid of *Kcnip2*^{GlyT2}-tdTom neurons. Thus, Cre-mediated recombination in *Kcnip2*^{roxCre}*;GlyT2::Dre* mice was found to be mostly restricted to the spinal cord and brainstem, while it was completely absent from DRGs and forebrain areas. Scale bar, 500 µm.

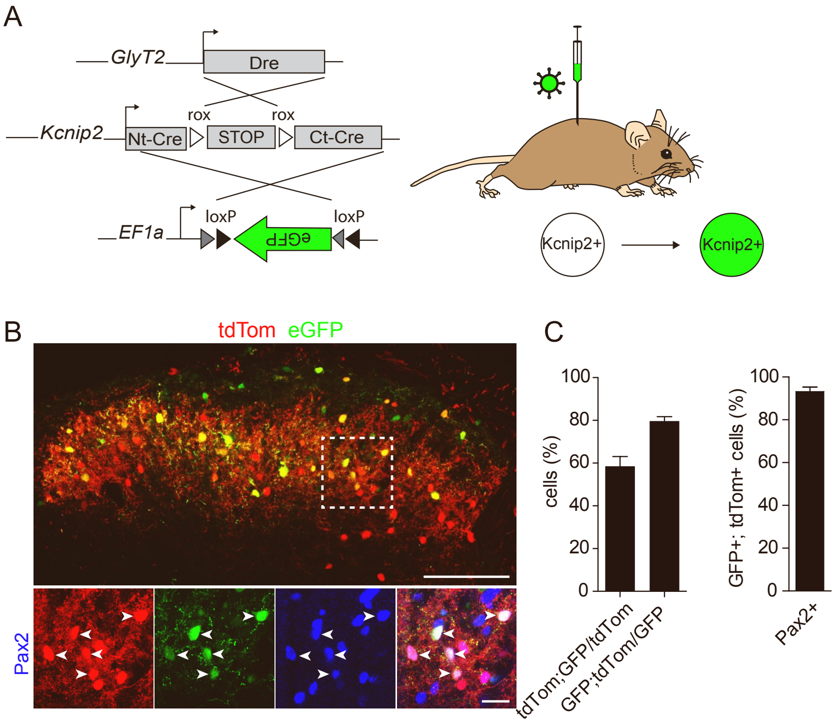

Fig. S6: Efficiency of intersectional AAV-mediated transgene expression in *Kcnip2*^{GlyT2} neurons, Related to Figure 3. A, *Kcnip2*^{GlyT2};*ROSA26*$^{lsl\text{-}tdTom}$ were intraspinally injected with an AAV carrying a cre-dependent eGFP expression cassette. **B**, transverse section of the lumbar spinal cord stained with antibodies against tdTom and eGFP. Scale bars, 100 µm (overview) and 20 µm (high magnification images). **C**, Quantification of co-expression of tdTom with eGFP. n = 12 sections from a total of three mice. Mean ± SEM.

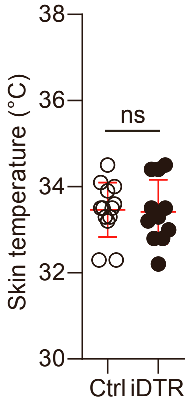

Fig. S7: Skin temperature of *Kcnip2*^{GlyT2} ablated mice, Related to Figure 3. Skin temperature was measured seven days after systemic (intraperitoneal) injection of diphtheria toxin (100 ng/g body weight). n = 11 control mice and n = 9 iDTR mice; ns, not significant (*P* = 0.88), unpaired t test. Mean ± SEM.

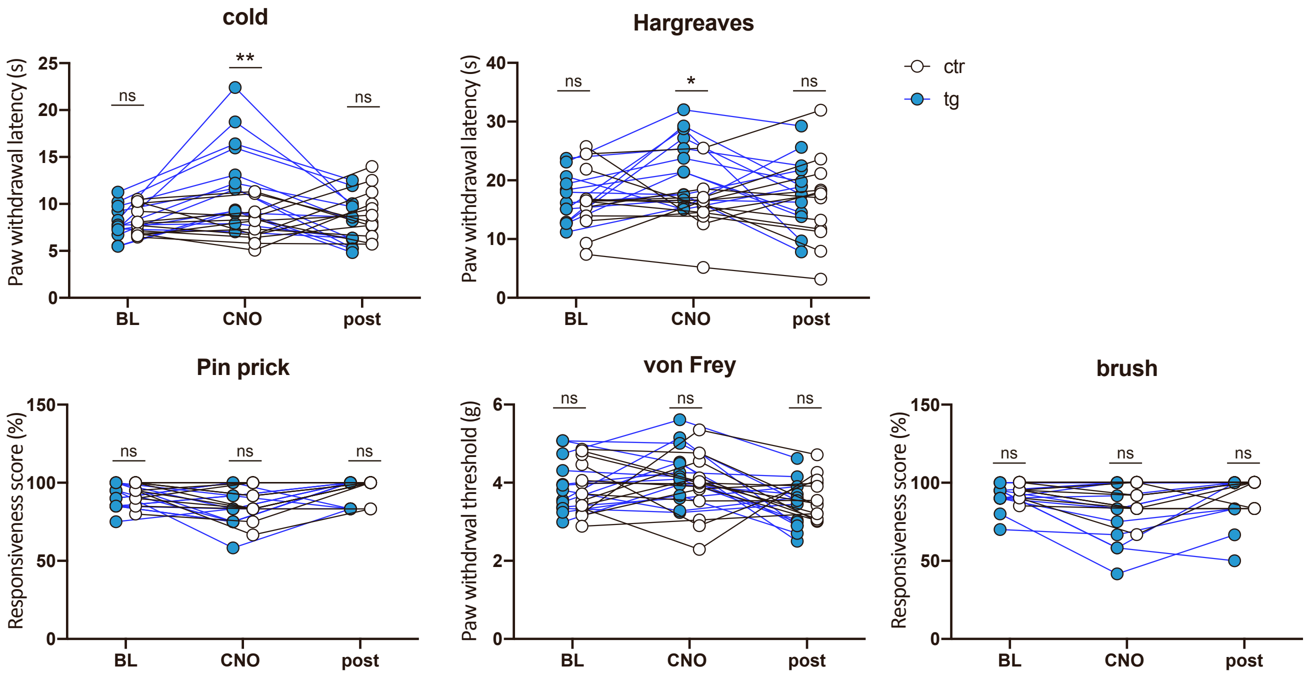

Fig. S8: Time course and reversibility of chemogenetic *Kcnip2*^{GlyT2} neuron activation, Related to Figure 4. Control (ctr) and *Kcnip2*^{GlyT2} (tg) mice were injected with an an AAV carrying a cre-dependent hM3Dq expression cassette. Mice were tested 24 h before CNO injection (BL), 1 – 2 hrs after CNO injection (CNO) and 24 h after CNO injection (post). Two-way ANOVA followed by Bonferroni correction**:** cold F(2,46) = 13.72, *P* < 0.0001; control versus tg in the presence of CNO: *P* = 0.0096; Hargreaves F(2,46) = 3.625, *P* = 0.0345; control versus tg (CNO), *P* = 0.0100; pin prick F(2,48) = 0.2406, *P* = 0.7871; control versus tg (CNO): *P* = 0.6258; von Frey F (2,48) = 1.442, *P* = 0.2465, control versus tg (CNO), *P* = 0.6656; brush F(2,48) = 0.8631, *P* = 0.4283, control versus tg (CNO), *P* = 0.3641. *, *P* < 0.05; **, *P* < 0.01; ns, not significant (*P* > 0.05). n = 13 for both genotypes.

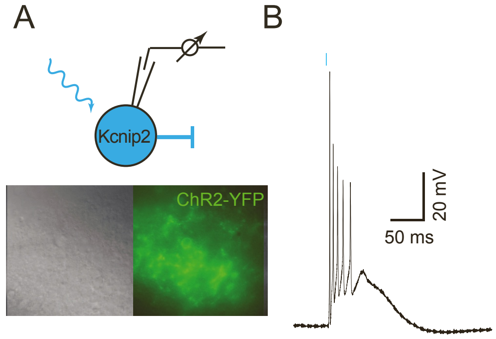

Fig. S9: Optogenetic stimulation of dorsal *Kcnip2*^{GlyT2} neurons, Related to Figure 6. A, Transverse spinal section. Left: IR gradient contrast, right, ChR2-YFP fluorescence. **B**, current clamp recording of a *Kcnip2*^{GlyT2} neuron. Blue line indicates blue light exposure.
